# Both semi-dwarf and photoperiod-insensitive traits in rice were important for the Green Revolution

**DOI:** 10.1186/s40529-026-00493-3

**Published:** 2026-03-26

**Authors:** Cheng-Chieh Wu, Yi-Tzu Tseng, Lin-Tzu Huang, Chun-Kai Liu, Kai-Cheng Hsu, Hao-Ching Wang, Yue-Ie Caroline Hsing, Wan-Yi Chiou

**Affiliations:** 1 Institute of Plant and Microbial Biology, Academia Sinica, Taipei, Taiwan; 2 Graduate Institute of Medical Genomics and Proteomics, National Taiwan University, Taipei, Taiwan; 3 Institute of Plant Biology, National Taiwan University, Taipei, Taiwan; 4 Graduate Institute of Cancer Biology and Drug Discovery, College of Medical Science and Technology, Taipei Medical University, Taipei, Taiwan; 5 Graduate Institute of Translational Medicine, College of Medical Science and Technology, Taipei Medical University, Taipei, Taiwan; 6 Department of Agronomy, National Chung-Hsing University, Taichung, Taiwan; 7 Present Address: Transgenic Plant Core Facility, Agricultural Biotechnology Research Center, Academia Sinica, Taipei, Taiwan

**Keywords:** Heading date, Indels, Natural variation, Rice, Semi-dwarf, SNPs, Protein structure

## Abstract

**Background:**

The semi-dwarf high-yielding rice (*Oryza sativa*) variety IR8 was the major modern line that gave rise to the “Green Revolution” in Asia approximately 60 years ago. Although the *sd1* mutation in IR8 responsible for semi-dwarfism is well known, the sequence variation, evolutionary origins, and functional impacts of *SD1* haplotypes remain incompletely characterized.

**Results:**

By analyzing whole-genome sequences from the 3K Rice Genomes Project, Taiwanese landraces, and Asian wild rice, we classified *SD1* into 12 haplotypes, including two newly identified loss-of-function alleles: a 1,279-bp deletion and an E100/R340 variant. Phylogenetic and nucleotide analyses traced the origins of several haplotypes, including the widely used 383-bp deletion (*DGWG* type), to the wild accession W1718 in southern China, from which they were later introgressed into landraces and subsequently brought to Taiwan. Protein structural modeling further revealed that distinct amino acid substitutions affect GA20ox-2 activity by altering the catalytic pocket or local protein stability. Screening of *SD1* and *Hd1* genotypes showed that most modern *indica* varieties carry loss-of-function alleles of both *sd1* and *hd1*, reflecting strong post–Green Revolution selection for semi-dwarfism and early maturity.

**Conclusion:**

Our results clarify the evolutionary history and functional consequences of *SD1* variation and highlight that the combined loss-of-function *sd1–hd1* genotype was central to the development of high-yielding, lodging-resistant, and photoperiod-insensitive rice varieties. These findings provide a comprehensive framework for understanding key Green Revolution genes and offer guidance for future rice improvement and genome-editing strategies.

**Supplementary Information:**

The online version contains supplementary material available at 10.1186/s40529-026-00493-3.

## Background

The “Green Revolution” of the 1960s led to a great increase in global production of rice (*Oryza sativa*) (Hargrove and Cabanilla 1979; Khush 1999) and wheat (*Triticum aestivum*) (Chapman et al. 2007). The newly developed high-yielding varieties of these two important staple crops fed the rapidly expanding world population. The key trait contributing to this higher productivity in both rice and wheat was the “semi-dwarf” trait, which is associated with the plant hormone gibberellic acid (GA) and reduces culm length without compromising yield. The new semi-dwarf varieties could withstand heavy winds and rainfall. In addition, they were resistant to lodging following treatment with a large amount of nitrogen fertilizer (Khush 1995).

The key Green Revolution variety of rice was IR8, also known as miracle rice; its *semi-dwarf 1* gene (*sd1*) encodes GA_20_ oxidase-2 (GA_20_ox-2; Os01t0883800) (Sasaki et al. 2002; Ashikari et al. 2002; Spielmeyer et al. 2002). The parental lines of IR8 were Dee-Geo-Woo-Gen (DGWG), a Taiwanese native semi-dwarf *indica* landrace, and Peta, a tall Indonesian *indica* landrace known for its high eating quality. IR8 was bred by the International Rice Research Institute (IRRI) in late 1966 (Hargrove and Cabanilla 1979). Both IR8 and DGWG contain a 383-bp deletion in *GA*_*20*_*ox-2* (*sd1*), affecting parts of exons 1 and 2, that leads to the loss of function (LOF) of this gene (Sasaki et al. 2002; Ashikari et al. 2002; Spielmeyer et al. 2002).

Single-nucleotide polymorphisms (SNPs) also occur in other *sd1* mutants. The Japanese native variety Jikkoku has a Gly (G) 94 to Val (V) substitution (Kikuchi et al. 1985). Two semi-dwarf varieties developed by γ-ray radiation have SNPs in *sd1*: Reimei has an Asp (D) 349 to His (H) substitution from the *japonica* cultivar Fujiminori (Futsuhara et al. 1967), and Calrose 76 has a Leu (L) 266 to Phe (F) substitution as compared to the US *japonica* cultivar Calrose (Foster and Rutger 1978). The SNPs in the *GA20ox-2* gene in these accessions thus represent three independent alleles leading to the semi-dwarf phenotype (Sasaki et al. 2002; Ashikari et al. 2002). A survey of additional semi-dwarf rice accessions revealed further alleles, including a 2-bp deletion causing a frameshift at amino acid (aa) 130 in Aijiao-Nante (*indica*); an SNP in Zhayeqing 8 (*indica*) resulting in a substitution of Pro (P) 240 to Leu (L); and another SNP in 9311 (*indica*), replacing Tyr (Y) 342 with a stop codon and causing early translational termination (Tyr342*) (Asano et al. 2007). More recently, a new *sd1* allele in Pusa 1652, a γ-ray–induced mutant of the short-grain aromatic landrace Bindli in India, was reported to introduce a premature stop codon (Tyr300*) (Bhuvaneswari et al. 2020).

The *SD1* gene has been revealed to be a target of selection during early rice domestication. Quantitative trait locus (QTL) mapping was conducted to identify the genetic basis of culm length using backcross inbred lines derived from a cross between the *japonica* rice variety Nipponbare and the *indica* variety Kasalath (Asano et al. 2011). High-resolution mapping with approximately 5,000 plants segregating at the *qCL1* locus indicated that the target gene was *SD1*, and that the functional nucleotide polymorphisms corresponded to amino acid substitutions: Glu (E) and Gln (Q) at residues 100 and 340 in Nipponbare, but G and Arg (R), respectively, at the same residues in Kasalath. The *EQ* variant of the double polymorphism exhibits lower enzymatic function. Studies focusing on landraces indicated that the *GR* haplotype is present in most *indica* and wild rice accessions, whereas *EQ* is predominant in *japonica* rice (Asano et al. 2011). A recent report indicated that the *GR*-*EQ* SNPs have existed in ancient rice populations since before domestication (Alam et al. 2025).

An analysis of the genealogy of the rice Green Revolution gene, including the *DGWG* and *Jikkoku* alleles (Nagano et al. 2005), suggested that the *DGWG* and *Jikkoku* alleles derive from different lineages. By examining the 383-bp deletion, the authors determined that the *DGWG* allele was present in several lines, including the IRRI *indica* varieties IR8 and IR36, the Taiwanese landrace Liu-T’ou-Tu, and two wild rice (*O. rufipogon*) accessions collected in southern China: W1718 and W1944. Because these wild rice accessions have unique polymorphisms in the region surrounding the *SD1* locus, the authors suggested that the *DGWG* allele might have originally been present in the wild progenitors rather than being introgressed from high-yielding varieties into the nearby wild rice (Nagano et al. 2005).

GA_20_ox-2 catalyzes the late steps of GA biosynthesis (Ashikari et al. 2002; Spielmeyer et al. 2002; Pimenta Lange and Lange 2006). This enzyme is responsible for the early 13-hydroxylation pathway of GA biosynthesis in vascular plants (Toyomasu et al. 1997; Spielmeyer et al. 2002; Pimenta Lange and Lange 2006), which starts with the transformation of GA_53_ to GA_44_, GA_19_, and GA_20_, ultimately yielding the bioactive form GA_1_. That is, SD1 is involved in the reaction of GA_12_ and GA_53_ to generate the bioactive GA precursors GA_9_ and GA_20_. The contents of individual GA compounds in equivalent elongating stem segments vary between tall and semi-dwarf rice plants, including Kyeema (tall, *SD1*) versus Doongara (dwarf, *sd1*) and Calrose (tall, *SD1*) versus Calrose76 (dwarf, *sd1*) (Spielmeyer et al. 2002). In particular, the content of GA_53_, the initial substrate of GA_20_-oxidase activity, is significantly higher in the semi-dwarf rice varieties (Doongara and Calrose76) than in their tall counterparts. Additionally, the contents of GA_20_, the major product of the pathway, and bioactive GA_1_ are lower in the semi-dwarf varieties than in the other varieties (Spielmeyer et al. 2002).

In the IR8 breeding history reports, the IRRI provided a blueprint for the variety in the 1961–62 Annual Report: “*a combination of short*,* stiff culms bearing erect*,* moderately sized*,* dark-green leaves; responsiveness in yield to fertilizer; mid-season maturity and in most cases*,* photoperiod sensitivity to permit double cropping practices.*” And, in 1963, they performed selection of F_2_ plants from the Peta–DGWG cross in which “*only short*,* early maturing plants were saved*” (Khush et al. 2001; Hargrove and Coffman 2006). A few key genes are thought to affect rice sensitivity to photoperiod, such as *Heading date 1* (*Hd1*), which encodes a B-box zinc finger protein and is the ortholog of Arabidopsis (*Arabidopsis thaliana*) *CONSTANS* (*CO*) (Yano et al. 1997, 2000); *Hd3a*, which encodes a phosphatidylethanolamine binding protein and is the ortholog of Arabidopsis *FLOWERING LOCUS T* (*FT*) (Tamaki et al. 2007); and *Grain number*,* plant height*,* and heading date 7* (*Ghd7*), an important regulator of heading date and yield potential (Xue et al. 2008). More than 10 loss-of-function alleles of *hd1* have been reported (Takahashi et al. 2009; Yano et al. 2000; Doi et al. 2004; Wu et al. 2020).

In the present study, we explored the mutation types of *SD1* and their evolution in rice by analyzing changes in the *SD1* sequence using the 3000 (3K) Rice Genome sequence database. Together with the 10 haplotypes discovered previously, we summarized 12 haplotypes, some of which have been used extensively in modern breeding. We studied the possible origins of some of these mutations. Using information about amino acid changes associated with the semi-dwarf trait, we also performed structural predictions to evaluate the effects of these changes on GA_20_-oxidase activity. We further suggest that the photoperiod-insensitive trait from DGWG also played an important role in the Green Revolution. Our findings shed light on the origins of the semi-dwarf and photoperiod-insensitive traits of rice that emerged during the Green Revolution.

## Results

### Many *sd1* haplotypes are present in rice accessions

**Fig. 1:**
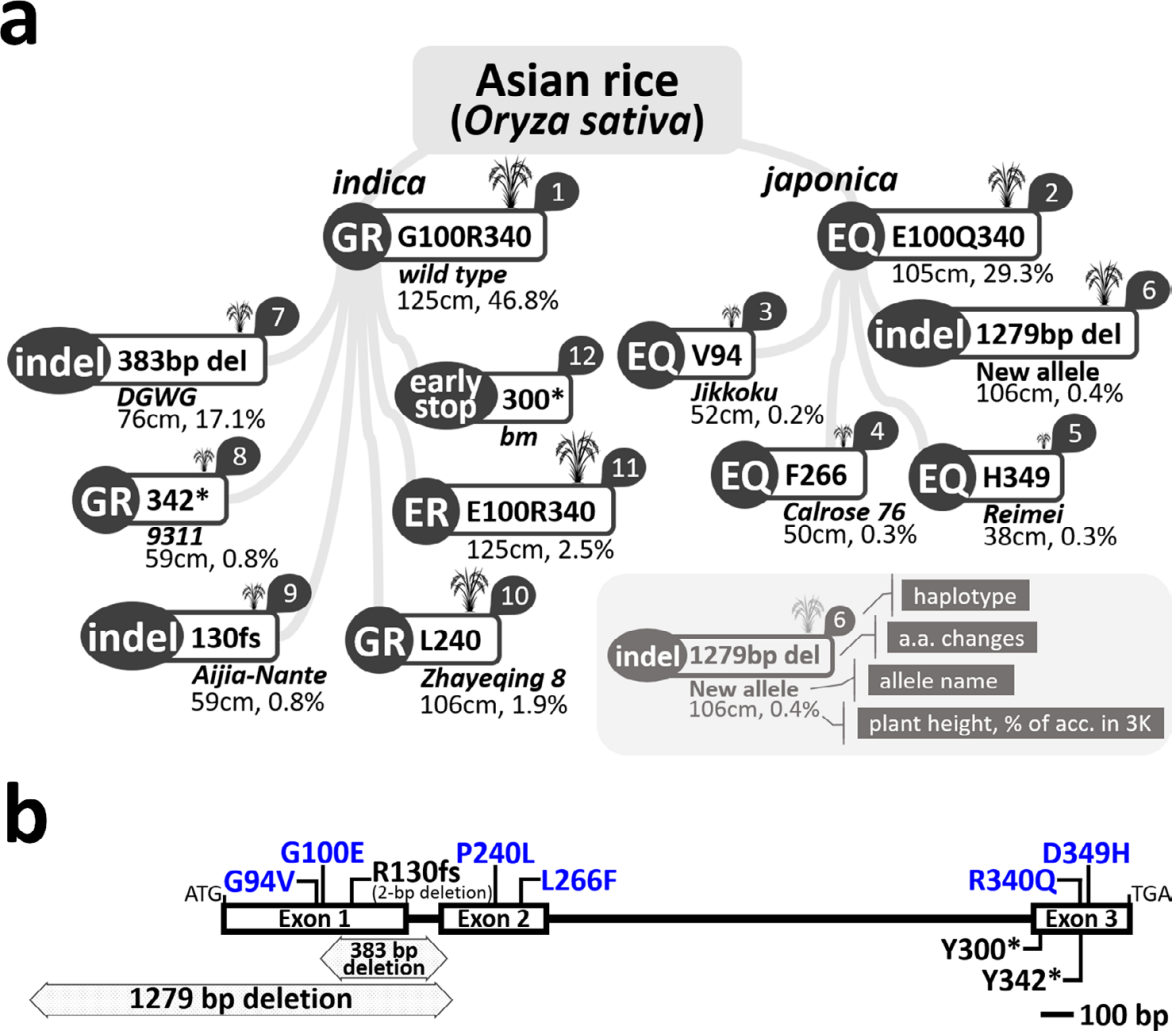
Overview of *SD1* haplotypes and mutation sites in rice. (**a**) Schematic representation of the 12 *SD1* haplotypes identified in this study. Haplotypes are grouped according to subspecies (*indica* and *japonica*). Each type is labeled with the corresponding amino acid change or deletion, allele name, average plant height, and frequency in the 3K Rice Genome dataset. GR represents the *wild type* allele (G100/R340), whereas EQ and ER denote alternative combinations at residues 100 and 340. Indel mutations and premature stop codons are indicated. (**b**) Gene structure of SD1 (*GA*_20_*ox*-2) and positions of mutation sites. The *SD1* gene contains three exons and two introns. The deleted sequences in the type 6 and 7 *SD1* haplotypes are represented by horizontal gray bars. The amino acid changes caused by single-nucleotide polymorphisms or short deletions of type 2–5 and 8–10 *SD1* haplotypes are shown. Amino acid substitutions are in blue. Bar, 100 bp

In the current study, we discovered previously unknown null *sd1* alleles from the 3K Rice Genome sequencing data (3K-RGP 2014), a 1,279-bp deletion variant lacking the promoter, exon 1, and intron 1, and an E100/R340 (ER) variant in addition to the previously described *GR* and *EQ*. Based on this information and the previously discovered haplotypes, we classified the *SD1* gene into 12 haplotypes (Table 1 and S1). Using the 3K dataset and the wild rice pan-genome data (Zhao et al. 2018), we confirmed that *GR* is present in most *indica* and all wild rice accessions, while *EQ* is present in most *japonica* accessions (Table 2). Thus, we defined *GR* as the type 1 (wild type) *SD1* haplotype (Fig. 1a). There were no *SD1-GQ*-type alleles (another combination of G/E and R/Q) in the natural population.

**Table 1:** Haplotypes of *SD1*

Haplotype	Allele name	Amino acid changes	Position(s) on chromosome 1	Number of accessions in the 3K dataset^a^	References
1	*GR*	Gly100 and Arg340	38,382,764 and 38,385,057	1235	Asano et al. 2011
2	*EQ*	Glu100 and Gln340	38,382,764 and 38,385,057	773	Asano et al. 2011
3	*Jikkoku*	Gly94Val	38,382,746	4	Sasaki et al. 2002
4	*Calrose76*	Leu266Phe	38,383,363	7	Sasaki et al. 2002; Spielmeyer et al. 2002
5	*Reimei*	Asp349His	38,385,083	9	Sasaki et al. 2002
6	New allele	1279-bp deletion	38,381,879–38,383,157	10	Current study
7	*DGWG*	383-bp deletion	38,382,762–38,383,144	450	Sasaki et al. 2002
8	*9311*	Tyr342* (early stop)	38,385,064	21	Asano et al. 2007
9	*Aijiao-Nante*	Arg130fs (2-bp insertion)	38,382,846–38,382,847	15	Asano et al. 2007
10	*Zhayeqing 8*	Pro240Leu	38,383,286	50	Asano et al. 2007
11	*ER*	Glu100 and Arg340	38,382,764 and 38,385,057	65	Current study
12	*bm*	Tyr300* (early stop)	38,384,938	N/A	Bhuvaneswari et al. 2020
Total				2639	

^a^Includes only accessions with high sequence quality (reads with quality ≥ Q20). Accessions carrying only type 1 or type 2 mutations were identified first; type 3–12 mutations were classified separately because these accessions may share the same amino acid substitutions at positions 100 or 340 as type 1 or 2 mutations. *, termination codon. fs, frame shift.

**Table 2:** Percentages of *GR*,* EQ*, and *ER* at aa positions 100 and 340 in the 3K panel^a^

	*indica*	*aus*	admix	*japonica*	*aromatic*
G100/R340	80.7	73.8	61.9	0	17.9
E100/Q340	0.8	1.3	19.0	89.0	82.1
E100/R340	5.9	16.3	4.8	0.7	3.4

^a^Only traditional landraces in the 3K panel were used for analysis, and only homologous alleles were counted

The remaining *sd1* haplotypes were categorized as SNPs, short indels, or long deletions. As compared with the wild type, types 2, 3, 4, 5, 8, 10, 11, and 12 had SNPs causing non-synonymous aa residue changes or early stop codons (i.e., E100/Q340, G94V, L266F, D349H, Y342*, P240L, E100/R340, and Y300*, respectively). Type 9 contained a 2-bp indel that led to a frameshift at aa position 130. The 383-bp deletion of the type 7 *sd1* haplotype abolished parts of exons 1 and 2 as well as intron 1. The type 6 haplotype lacked the promoter region, exon 1, and intron 1 due to a 1,279-bp deletion (Fig. 1b). Integrative Genomics Viewer (IGV) snapshots of the 383- and 1,279-bp deletions in *SD1* showed that their 3′ ends were only 13 bp apart—at positions 38,383,145 and 38,383,158 of chromosome 1, respectively (Fig. S1)—although their 5′ ends were separated by ~ 900 bp.

**Fig. 2:**
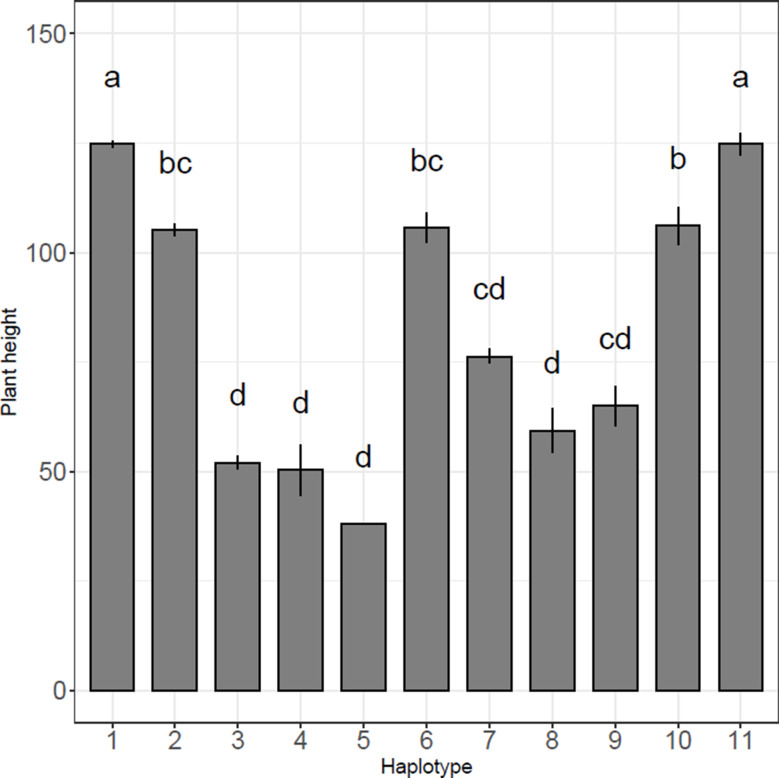
Plant height of the 11 *SD1* haplotypes. Plants were grown at the International Rice Research Institute, and plant height (culm length) was measured during the heading period. The phenotype information was downloaded from the SNP-SEEK website (https://snp-seek.irri.org/). Sample number and detailed statistical data are shown in Table 1, Table S2, and Table S3. Type 1 is the wild type (*GR* type). Data are mean ± SD. Different letters above the bars indicate significant differences at *P* < 0.05

To evaluate the phenotypic impact of these diverse *sd1* haplotypes, we analyzed plant height data from the IRRI SNP-SEEK phenotype database (https://snp-seek.irri.org/) (Mansueto et al. 2017; Alexandrov et al. 2015). This database provides plant height information for rice cultivated at the IRRI, alongside extensive phenotypic data for approximately two-thirds of the 3K accessions. Data on culm length at heading were retrieved from the database and grouped by *SD1* haplotype. Accessions excluding types 3 through 10 were categorized as *GR*, *EQ*, or *ER*. Analysis of variance (ANOVA) revealed significant variation in plant height among the *sd1* haplotypes (Table S2). Multiple comparisons demonstrated that plants carrying types 2–10 differed significantly in height from the wild type (type 1) (Fig. 2 and Table S3). The significant height reduction in plants with type 2, which lacked other structural mutations, confirmed that the *GR*-to-*EQ* substitution alone represents a mutation with reduced function, exerting a clear effect on plant height at heading. In contrast, type 11 (carrying the *ER* allele) exhibited the same plant height as the wild type, indicating that this mutation did not alter plant stature.

To explore the presence of *GR* and *EQ* and even the intermediate allele *ER* in natural rice populations, we investigated the variants of the *SD1* gene present in the 3K panel. Only homozygous SNPs were counted. No *japonica* accessions and six *aromatic* accessions featured the *GR* type; approximately 40 *indica* and *aus* accessions featured the *EQ* type. Details about the total number of accessions and traditional landraces with these alleles are shown in Table S4. Notably, some rice accessions harbored the *SD1-ER* allele (type 11): specifically, 6 *japonica*, 2 *aromatic*, 40 *aus*, and 154 *indica* accessions. These alleles were quite rare in landraces: 2 were found in *japonica*, 1 in *aromatic*, 13 in *aus*, and 38 in *indica* rice.

Accessions with type 3–5 mutations were the shortest plants among all groups. These were semi-dwarf, less short than previously reported dwarf mutants (Futsuhara et al. 1967; Foster and Rutger 1978; Kikuchi et al. 1985). However, type 3–5 plants grown in the IRRI paddy field were considerably shorter than other *sd1* mutants (types 6–10). There were likely several factors contributing to these height differences. Plant height was measured at 80% heading. Since photoperiod sensitivity determines the number of days to heading, the temperate *japonica* lines grown in tropical regions headed significantly earlier (~ 2 months after planting) than most other *sd1* mutant types (at least 3 months), which directly led to a shorter final plant height (Fig. 2 and Table S3).

Beyond these physiological factors, it is important to note that potential inconsistencies in seed purity within germplasm collections could complicate further analysis. For instance, among the two Aijiao-Nante accessions sequenced in the 3K dataset, only IRIS_313-12273 had the type 9 (2-bp) deletion. The other accession (CAAS-B060) contained the 383-bp deletion (type 7). Similar discrepancies were observed in Taichung Native 1 (TN1). As the first modern semi-dwarf variety derived from a cross between DGWG and Tsai-Yuan-Chung, TN1 is expected to inherit the type 7 null allele from DGWG. However, of the two TN1 accessions in the 3K dataset, only CX162 carried the 383-bp deletion, while CX270 did not. Furthermore, although the famous Japanese variety Koshihikari is known to lack the 383-bp deletion (Tomita 2009), the 3K accession CAAS_CX330 was unexpectedly grouped into *sd1* type 7.

### The origins of the *SD1* haplotypes

Our above results indicated that four *SD1* haplotypes were specific to *japonica* rice: types 3, 4, 5, and 6. Types 3 and 5 corresponded to *Jikkoku* and *Reimei* from Japan, respectively. Type 4 was *Calrose 76* from the United States. Ten *japonica* accessions carried *sd1* type 6, which harbored a 1,279-bp deletion. Plotting the geographic origins of type 6 accessions using GPS information (Gutaker et al. 2020) showed that they were all collected in mountainous regions of Laos and Thailand (Fig. S2). To determine whether haplotypes with the longer deletion (type 6) were derived from those with the shorter deletion (type 7), we performed phylogenetic analysis of the upstream and downstream 1-Mb regions of *SD1*. Types 6 and 7 alleles were in different clusters (with the former including only *japonica* accessions and the latter primarily *indica* accessions; Fig. S3; accessions are listed in Table S5). In addition, a SNP (C to T at position 38,383,221) was consistently associated with accessions containing the 383-bp deletion but not the 1279-bp deletion. Hence, we concluded that these two alleles were derived from different lineages. Phylogenetic analysis of the ± 1-kb region of *SD1* indicated that these type 6 lines shared an extremely close relationship (Fig. S4, accessions listed in Table S6), which suggests that the mutation occurred recently. Accessions with this allele were tropical *japonica* accessions collected from part of the Indochina peninsula.

There were four types of *sd1* mutations in *indica* rice. The *9311* mutation (type 8) occurred *in agro* in China. *Aijio-Nante* (type 9) harbored a mutation that also occurred *in agro* in the cultivar Nante in China; notably, the name Aijio is translated as low foot (semi-dwarf). Table S1 shows that 50 accessions contained the *Zhayeqing 8 sd1* mutation (type 10); 21 were traditional landraces. Given that more than 95% of the traditional landraces were collected from Indonesia and Malaysia, and that phylogenetic analysis of the ± 1-kb region of *SD1* indicated that the Indonesian lines were closely related to wild rice accessions (Fig. S5, accessions listed in Table S7), we conclude that type 10 arose in the insular region of Southeast Asia.

DGWG, which has the first null *sd1* allele (type 7) identified, was a Taiwanese *indica* landrace. A previous study analyzing polymorphisms of three molecular markers (KS3, RM8278, and RM1387), located approximately 6–7 cM from the *SD1* locus, suggested that this mutation first arose in wild rice in southern China and was later introgressed into local landraces or varieties (Nagano et al. 2005). Nagano et al. (2005) also noted that the Taiwanese landrace Liu-T’ou-Tu carries DGWG allele. Thus, we searched for accessions with the *DGWG* allele in the 3K panel and found that another Taiwanese landrace, Hsinchu-Ai-Chueh-Chien, had the same deletion. Through detailed data mining of information about landrace collections in the National Plant Genetic Resources Center (NPGRC, https://www.npgrc.tari.gov.tw), Taiwan Agricultural Research Institute (TARI), we discovered at least three additional semi-dwarf lines, including Ti-Chueh-Wu-K’o, Ai-Tzu-Ch’ung, and Liu-Tou-Tzu (Table S8; Wu et al. 2022). According to historical records, these Taiwanese landraces were introduced from Guangdong during the migration of Han people to Taiwan in the Ming and Qing dynasties, primarily between the 17th and 19th centuries (DAFTPG 1989). We confirmed that Liu-T’ou-Tu, described in Nagano et al. (2005), was identical to Liu-Tou-Tzu in the NPGRC collection but had its name misspelled. In that study, the two wild rice accessions carrying the *DGWG* mutation were determined to be W1718 and W1944, both from Guangdong, southern China. However, based on downloaded sequencing information (Zhao et al. 2018), only W1718, but not W1944, carried the 383-bp deletion. This may be another example of seed mixing in the stock center.

**Fig. 3:**
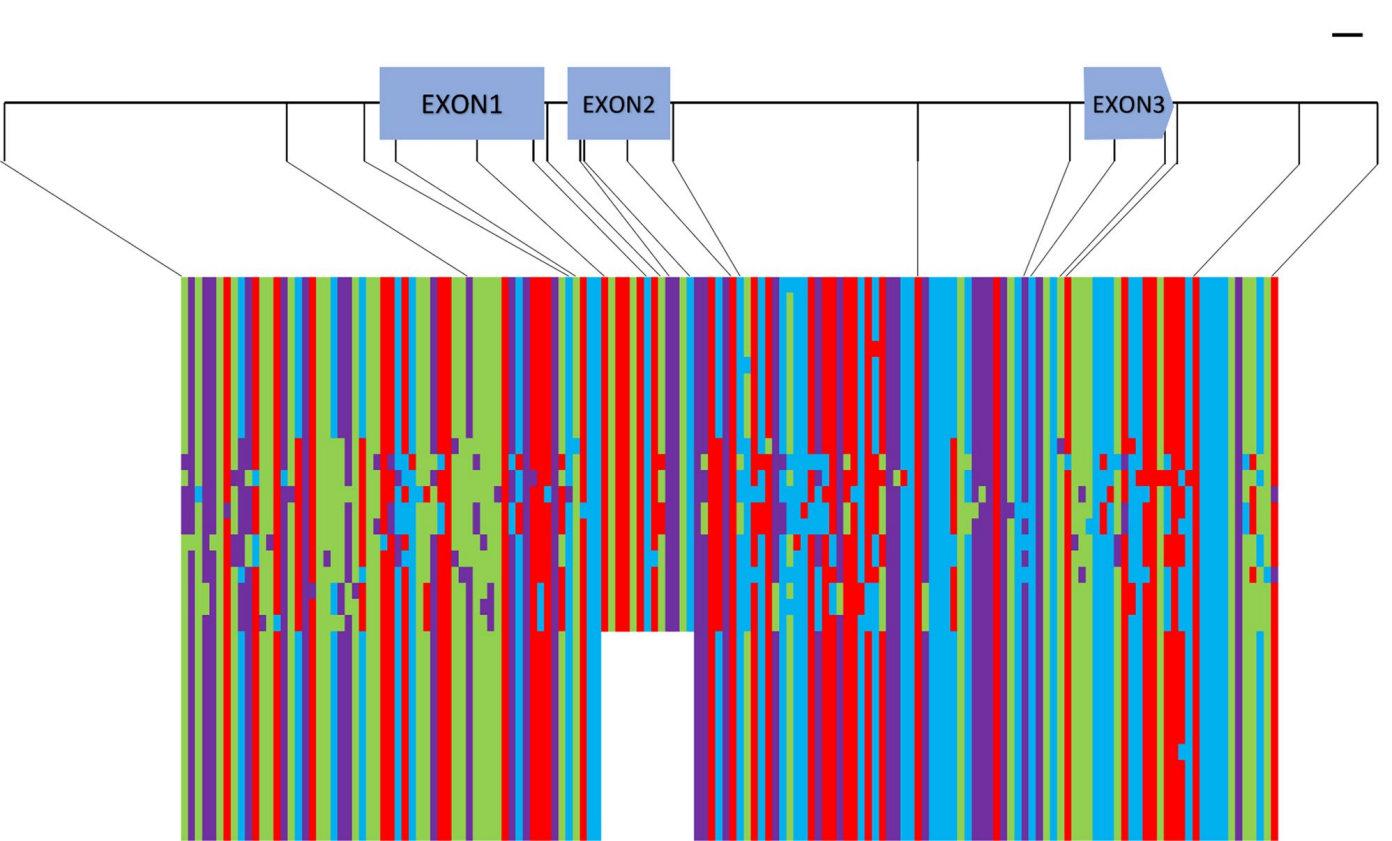
Haplotype display of *sd1* type 7. The single-nucleotide polymorphisms (SNPs) from position − 1,000 to position + 1,000 bp of the gene were plotted. The blue bars at the top indicate three exons. Purple, A; blue, T; green, G; red, C. Accessions are as follows (listed from top to bottom): 10 Taiwanese *indica* landraces: Taichung Woo Gen 2, Chiayi Wu-K’o, Taitung Woo-Li, Ti-Chueh-Hua-Lou, Ti-Chueh-Liu-Chou, Ti-Chueh-Ko-Tzu, Hua-Lou, Wu-Chan, Wu-K’o, Ai-Chueh-Ching-Yu; 13 wild rice accessions: W0123, W1739, W1754, W1777, W2012, W0141, W0170, W1698, W1979, W1943, W3078, W3095, W1718; and 12 accessions with the 383-bp deletion: Taichung Native 1, Hsinchu-Ai-Chueh-Chien, Dee-Geo-Woo-Gen, Ti-Chueh-Wu-K’o, Ai-Tzu-Chung, Liu-Tou-Tzu, Taichung Sen Yu 214, Wagwag Puti, IR64, IR 5494, X23, NON HAI. Information about the collection regions and DNA accession numbers is provided in Table S4

To reveal the origin of the 383-bp deletion and its presence in wild rice accessions, we chose the five Taiwanese landraces with the type 7 *sd1* mutation, along with other landraces, modern varieties, and wild rice accessions, for further analysis. Figure 3 shows the SNPs from position − 1073 to + 1199 bp around the *SD1* gene in 35 accessions, including landraces or modern varieties with or without the type 7 mutation, as well as 13 wild rice accessions (samples listed in Table S9). The SNP patterns of the 12 landraces and modern varieties with the 383-bp deletion, along with the wild rice accession W1718, are basically identical in the entire 2.7-kb region (including the promoter, three exons, and two introns, as well as the 5′ and 3′ untranslated regions). In addition, in the region excluding the 383-bp deletion fragment, the 10 Taiwanese *indica* landraces without the type 7 mutation, such as Wu-Chien, Wu-K’o, and Wu-Li, share a pattern similar to that of the landraces and W1718, which contain the 383-bp deletion. However, 12 other wild rice accessions have diverse SNP patterns in this 5-kb region. This suggests that W1718 must share a very close relationship with landraces and varieties with the 383-bp deletion.

To explore the lineage of the type 7 *sd1* mutation, we performed detailed phylogenetic analyses (Fig. S6; Table S10) of the ± 100-kb region (Fig. S6A), ± 9-Mb region (Fig. S6B), and ± 10-Mb region (Fig. S6C) around the *SD1* gene as well as the whole genome (Fig. S6D). The wild rice W1718 (highlighted in purple) was tightly clustered with accessions with the type 7 mutation (highlighted in red) in the ± 100-kb region (Fig. S6A); it remained in the clade with Wu-Chien, Wu-K’o, Wu-Li, and accessions with the type 7 mutation in the ± 9-Mb region (Fig. S6B). In the phylogenetic tree for the ± 10-Mb region (Fig. S6C), however, this wild rice was separate from the clade containing cultivars, and it was placed in the wild rice clade in the phylogenetic tree based on whole-genome analysis (Fig. S6D).

**Fig. 4:**
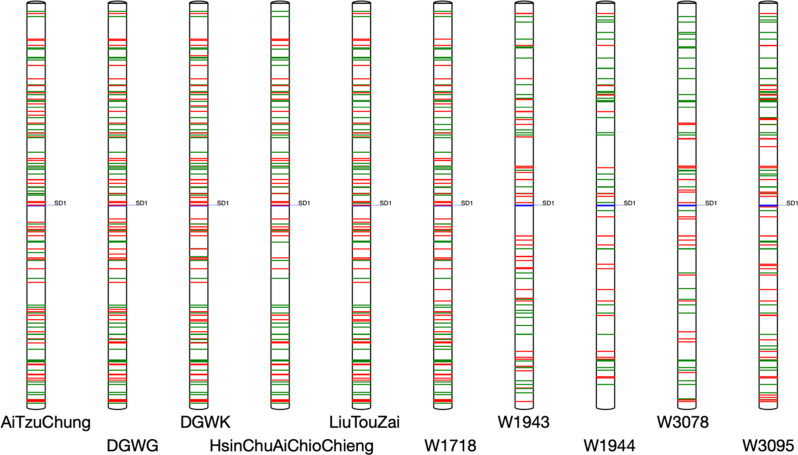
Distribution of tandem repeats (TRs) in the *SD1* gene region. The ± 1-Mb regions of *SD1* genes in five Taiwanese landraces with type 7 mutations and five wild rice accessions collected in southern China are shown. The accession names are indicated below. The positions of *SD1* genes are marked. Red bands indicate TRs found only in the current accession compared with the Nipponbare genome, and green bands indicate TRs not in the current accession (but detected in the Nipponbare genome). TRs ranging from 8 to 10 bp with 100% identity were included in this plot. Bar, 100 kb

To further confirm the lineage, we examined the localizations of the 8- to 10-bp tandem repeats (TRs) around the *SD1* gene applying a previously developed software package that quickly scans TRs and plots them along chromosomes (Wei et al. 2016). The genes of eukaryotes, including rice (IRGSP 2005), contain highly divergent TRs (Gemayel et al. 2010; Zhao et al. 2014). TRs occupy approximately 5% of the Nipponbare rice genome (Korotkov et al. 2021). For instance, there are 6083, 4925, and 4886 copies of 8-, 9- and 10-bp TRs, respectively, in the Nipponbare RefSeq data. In the ± 1-Mb *SD1* gene region, we assessed the patterns of TRs that were present only in the current accession but not in the Nipponbare genome, and TRs that were not present in the current accession (but were present in the Nipponbare genome). The patterns for the five Taiwanese landraces (Ai-Tzu-Ch’ung, DGWG, Ti-Chueh-Wu-K’o, Hsinchu-Ai-Chueh-Chien, Liu-T’ou-Tzu) and wild rice W1718 were very similar, whereas each of the four other wild rice accessions from southern China had a unique TR pattern (Fig. 4). Along with identical SNPs in the ± 1-kb region (Fig. 3), high sequence similarity around the end of the long arm of chromosome 1 was present in this specific wild rice accession a long time ago in Guangdong, southern China. These results suggest that the 383-bp deletion originated in the wild rice accession W1718 in southern China and was subsequently introgressed into neighboring landraces.

Our data suggest that the type 7 *sd1* allele occurred *in agro* at least 500 years ago in southern China, before the mass migration of the Han people to Taiwan (DAFTPG 1989). Consequently, when thousands of landraces were introduced to Taiwan during this migration, some already carried the type 7 mutation. In contrast, semi-dwarf landraces carrying the 383-bp deletion were not preserved in southern China, whereas wild rice accessions such as W1718, which were not subject to human selection, continued to propagate and retained this mutation. We explored which type 7 accession(s) were brought to Taiwan by analyzing the nucleotide variation in the ± 200-kb region surrounding *SD1* in five type-7 Taiwanese landraces and five wild rice accessions collected from Guangdong, China. Phylogenetic analysis placed Hsinchu-Ai-Chueh-Chien closest to wild rice (Fig. S7, accessions listed in Table S11). A Venn diagram of SNPs across the whole genomes of the five landraces revealed that DGWG and Hsinchu-Ai-Chueh-Chien possessed numerous unique SNPs (> 100,000), whereas the other three landraces had fewer unique SNPs (< 100,000) (Fig. S8). These results suggest that DGWG and Hsinchu-Ai-Chueh-Chien were the accessions most likely brought to Taiwan by Han migrants.

### A selective sweep occurred only for the E100/Q340 *sd1* mutation

To test early selection for the *EQ* allele, we analyzed traditional Asian *japonica* landraces that were homozygous for type 2 alleles. In total, 293 accessions were included, representing primarily temperate, subtropical, and tropical *japonica* from Asia (Table S12). Type 4 and type 5 mutations, which were induced by γ-ray treatment, were excluded from the analysis, as were type 8 and type 10 mutations that arose after World War II. Ten accessions carrying the type 6 allele originated from Laos and Thailand, whereas most accessions with the type 10 allele were from Indonesia and Malaysia. A relatively high number of landraces with types 6, 7, and 10 alleles were available and included in the analysis.

We assessed several parameters, including nucleotide diversity (*π*; Tajima 1983), Watterson’s estimator (*θw*; Watterson 1975), and Tajima’s *D* (Tajima 1989), in the ± 1-kb and ± 10-kb regions. Among the four allele types examined, only type 2 (the *EQ* allele) showed a significantly negative Tajima’s *D* (*P* < 0.001) (Table S13), suggesting an excess of low-frequency polymorphisms in this region. In contrast, Tajima’s *D* values for the other allele types were not significantly different from zero in the landrace population.

### Alignment and analysis of missense mutations reveal the important domain of GA_20_ oxidase

The previous results showed that different alleles affected plant height. We wanted to pinpoint the location of the mutation sites in the GA_20_ox-2 protein structure to understand their impact on protein function. Four *SD1* gene homologs are present in the rice genome: Os01g0883800 (*SD1*), Os05g0421900, Os03g0856700, and Os07g0169700. We generated a protein sequence alignment of the four transcripts, including the positions of GR/EQ as well as each of the missense mutations leading to the semi-dwarf phenotype (Fig. S9). We used the SD1 protein sequence from Kasalath instead of Nipponbare because we considered *indica* rice to be the wild type in this context. The locations of the type 3, 4, 5, and 10 LOF mutations were highly conserved. In addition, R340 of the low-functionality mutation (type 2) was conserved, but G100 was not: Os01t0883800 and Os05t0421900 had G at this position, whereas two other homologs had Asn (N). The aa changes in these positions, including those denoted 1, 2, 3, 4, and a (Fig. S9), all led to reduced plant height during heading.

We used a structural approach to explore the effects of GA_20_ox-2 mutations. Because no crystal structure of GA_20_ox-2 has been reported, its enzymatic function was inferred from pea (*Pisum sativum*) GA_20_-oxidase, which converts GA_19_ and 2-oxoglutarate into GA_20_ and succinate (García-Martínez et al. 1997). Molecular docking was performed to generate a GA_20_ox-2/GA binding model. The validity of the resulting models was assessed by comparison with relevant reference structures, including GA_2_ox3 (PDB ID: 6KU3; Takehara et al. 2020) and thebaine 6-O-demethylase (PDB ID: 5O7Y; Kluza et al. 2018). Based on these evaluations, a GA_20_ox-2/succinate/GA_20_ docking model was selected for further analysis. In addition, a GA_20_ox-2/GA_20_ binding model was generated using the GA_2_ox3/GA_4_ complex (PDB ID: 6KU3) as a template. GA_20_ox-2 and GA_2_ox3 share 42% sequence similarity, and both oxidases function in GA biosynthesis in the cytoplasm. Given this similarity in both sequence and biological function, the GA_20_ox-2 structure is likely to closely resemble that of GA_2_ox3.

**Fig. 5:**
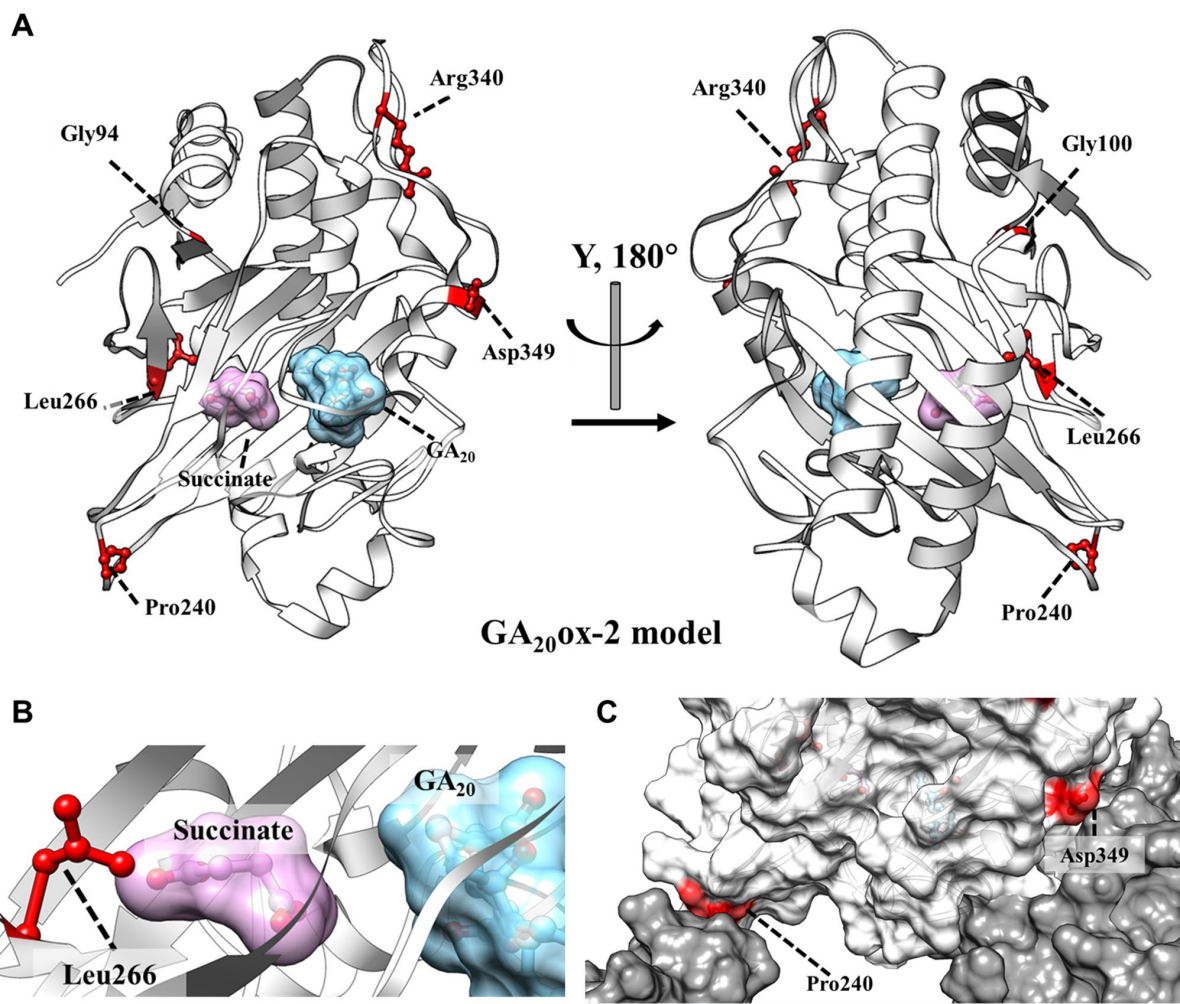
Model-based analysis of GA_20_ox-2 encoded by the *SD1* gene. (**A**) Model of two views of GA_20_ox-2 with all mutation sites labeled, generated using the MODELLER module. (**B**) The GA_20_ox-2 active site. The nearby location of Leu266 is indicated. (**C**) The positions of Asp349 and Pro240. The positions of the SD1 mutations in GA_20_ox-2 are shown in red

Referring to the GA_2_ox3 structure (Takehara et al. 2020), we generated a GA_20_ox-2 monomer model using the MODELLER module, in which all GA_20_ox-2 mutation sites were labeled (Fig. 5A). This analysis indicated that three of these sites may directly affect oxidase activity. The L266F mutation (type 4) occurred directly in the active site (Fig. 5B). Because phenylalanine has a larger side chain than leucine, this substitution may disturb the interaction between succinate and GA_20_. In contrast, D349 and P240 were located close to the interface regions of GA_2_ox3 (Fig. 5C). The D349H mutation (types 1 and 5) introduced a charge reversal, while P240L (types 1 and 10) increased hydrophobicity. Both mutations may disrupt intermolecular interactions by altering the local properties at these interfaces.

Apart from the three mutations described above, no further associations between GA_20_ox-2 activity and other mutations were evident from structural analysis. We hypothesize that mutations such as G100E and R340Q in type 2 variants may instead affect SD1 protein stability.

### *sd1* and *hd1* mutations are common in modern *indica* varieties

Before the Green Revolution, most rice landraces in Southeast Asia were tall, easily lodged, late maturing, and low yielding (Khush 1987; Khush et al. 2001; Gaur et al. 2020). In contrast, TN1 and IR8 are semi-dwarf and lodging resistant. Consequently, the *DGWG* type has been intensively utilized in rice breeding, accounting for 17% of the accessions in the 3K panel. The next most common was *Zhayeqing 8* (type 10) with 65 accessions (2.5%), while other types (3–6 and 8–9) comprised 66 accessions (Table 1). This suggests that the *DGWG* type was greatly favored by breeders to modulate plant height.

Another advantage of the modern varieties developed since the Green Revolution was their insensitivity to day length. To verify the genotypes of *SD1* and *Hd1* of major varieties in Green Revolution, we used the haplotypes defined by Wu et al. (2020) to screen the 3K dataset and several Taiwanese landraces. Table 3 lists the allele types of the *SD1* and *Hd1* genes in rice landraces associated with the Green Revolution. DGWG, IR8, Tsai-Yuan-Chung, and TN1 carry type 7 *hd1* alleles, as also reported by Panibe et al. (2021), whereas Peta carries the type 13 *hd1* allele.

**Table 3:** Haplotypes of *SD1* and *Hd1* genes in rice landraces related to the Green Revolution

	Origin	*SD1* haplotype	*Hd1* haplotype^a^
Hsinchu-Ai-Chueh-Chien	Taiwan	Type 7	Type 3
Ti-Chueh-Wu-Ko	Taiwan	Type 7	Type 7
Liu-Tou-Tzu	Taiwan	Type 7	Type 7
Ai-Tzu-Chung	Taiwan	Type 7	Type 7
DGWG	Taiwan	Type 7	Type 7
Peta	Indonesia	Type 1	Type 13
IR8	IRRI	Type 7	Type 7
Tsai-Yuan-Chung	Taiwan	Type 1	Type 7
Taichung Native 1	Taiwan	Type 7	Type 7

^a^*Hd1* haplotypes were defined by Wu et al. (2020)

We further assessed the distribution of *hd1* haplotypes among 3K dataset accessions harboring LOF *sd1* alleles, specifically quantifying those with *hd1* type 7 or type 13. This dataset includes information on more than 200 accessions each from three rice-growing countries—435 from India, 248 from Indonesia, and 229 from the Philippines—including traditional landraces, which were used for further analysis. To evaluate the importance of the LOF *hd1* allele in modern varieties, we calculated the ratio of non-traditional accessions to the total number of accessions for each country. The *hd1* type 7 allele (represented by the DGWG haplotype) showed an 83.02% increase in frequency from before to after the Green Revolution, in contrast to only a 20.0% increase for the type 13 allele present in Peta (Table S14). According to our previous studies of *Hd1*, both type 7 and type 13 mutations originated in insular Southeast Asia thousands of years ago (Wu et al. 2020). This result indicates that most modern *indica* rice varieties inherited both LOF *sd1* and *hd1* alleles from DGWG.

## Discussion

Before the Green Revolution, the plant height of most rice *indica* landraces at the maturing stage was over 160 cm (for example, 180–190 cm for Peta; Griffin 1979) and most were late maturing due to photoperiod sensitivity (Khush 1987). Since the introduction of the LOF *sd1* trait, breeders have intensively used *sd1* alleles to breed new varieties (Hargrove et al. 1988): for example, IR8 and IR64 in the Philippines (Khush et al. 2001) (labeled in yellow in Table S1) and Rasi and Jaya in India (Prasad et al. 2001). However, due to their partially functional *SD1-EQ* trait, most *japonica* landraces or modern varieties are 100–120 cm tall. *SD1*, one of the genes controlling rice plant architecture, has been an important target for early selection as well as modern breeding. Many alleles of the *sd1* mutants have been studied. In the present study, we classified the mutant alleles we detected into 12 types and explored the origin of each allele. Using the 3K dataset, we also revealed that all modern *indica* varieties but only a few modern *japonica* varieties harbor the mutated alleles. This distribution pattern reflects the intensive selection for both semi-dwarfism and photoperiod insensitivity that was fundamental to the success of the Green Revolution.

### Evolutionary and functional significance of the *SD1-EQ* allele in rice domestication and adaptation

At least 62 *dwarf* (*d*) and 15 *semi-dwarf* (*sd*) genes have been cataloged in rice (Kinoshita 1995), indicating that multiple genetic pathways can reduce plant height. However, *sd1* has been the principal semi-dwarf allele incorporated into modern breeding programs. Many other dwarf-related genes influence additional agronomic traits, such as tiller number, leaf greenness, leaf morphology, and spikelet number, causing undesirable pleiotropic effects. Within the GA_20_ oxidase family, functional differentiation is evident. *GA*_*20*_*ox-1* is broadly expressed and influences multiple traits, whereas *GA*_*20*_*ox-3* and *GA*_*20*_*ox-4* show tissue-specific expression in anthers and panicles, respectively. In contrast, *GA*_*20*_*ox-2* (*SD1*) is primarily expressed in the culm and mainly affects stem elongation without substantially altering other major agronomic traits (Sakamoto et al. 2004; Table S15). This tissue-preferential expression may explain why LOF *sd1* reduced plant height with relatively limited pleiotropic effects.

The *SD1-EQ* allele type was chosen and maintained during early *japonica* domestication (Asano et al. 2011; Ashikari et al. 2025; Alam et al. 2025). However, it was not introgressed into *indica* rice with other domestication traits, such as *non-shattering* (*sh4*; Li et al. 2006; Choi and Purugganan 2018) and closed stature (*prog 1*; Tan et al. 2008; Jin et al. 2008). Sanger sequencing of 72 landraces and 42 wild rice accessions revealed that the *EQ* type *SD1* gene was selected thousands of years ago in ancient *japonica* landraces, suggesting that *SD1* was involved in rice domestication (Asano et al. 2011). A comparison of GA_20_ oxidase activities of recombinant SD1-GR, SD1-EQ, SD1-GQ and SD1-ER proteins revealed that approximately three times more GA_20_ was produced by SD1-GR (wild type) than by the other three types, with no significant difference in GA_20_ production among SD1-EQ, SD1-GQ, and SD1-ER (Asano et al. 2011). The phenotypic records for these *SD1-ER* accessions showed that they had similar plant height to the wild type (Fig. 2, Table S3): that is, their heights did not correspond to the level of GA_20_ox-2 activity, as they did among *EQ* (type 2) accessions (Asano et al. 2011). This suggests that environmental conditions, particularly temperature and latitude, may modulate the phenotypic expression of the *GR* and *EQ* allele type.

The *EQ* allele has been proposed to have undergone preferential selection during *japonica* domestication approximately 5,000 years ago (Asano et al. 2011; Alam et al. 2025; Ashikari et al. 2025). In the 3K dataset, 89% of traditional *japonica* landraces are homozygous for the *EQ* allele, whereas approximately 81% of *indica* accessions contain a homozygous *GR* allele, and 12.6% are heterozygous (Table 2). These contrasting frequency patterns are consistent with differential historical processes acting on the *SD1* locus during rice domestication and subsequent breeding. Although demographic factors such as population bottlenecks cannot be excluded, the near fixation of the *EQ* allele in traditional *japonica* landraces, together with previous studies (Zhao et al. 2018; Kou et al. 2020), supports a scenario in which the *EQ* allele was established early during *japonica* domestication.

### Different spellings for the same lines may cause problems

Liu-Tou-Tzu was misspelled in another paper (Nagano et al. 2005), as noted previously. We noted other similar cases as well. “DG” in DGWG means “low foot (semi-dwarf),” and “WG” (Wu-Chien in NPGRC) means “black epiculus”; its sibling line Wu-Chien has a normal plant height. Another type 7 Taiwanese landrace, Ti-Chueh (semi-dwarf)-Wu-K’o (black glumes), also has a sibling line, Wu-K’o (with normal plant height). Both Wu-Chien and Wu-K’o are landraces whose genomes were sequenced in the 3K project. These two pairs of lines (i.e., DGWG and Wu-Chien; Ti-Chueh-Wu-K’o and Wu-K’o) were popular landraces cultivated in paddy fields in Taiwan during the Ching Dynasty (DAFTPG 1989). They are closely related to another sibling line, Wu-Li (black grain). Another semi-dwarf race, Hsinchu-Ai-Chueh-Chien, was also popular during the same period.

All these landraces were among the ~ 1000 rice lines brought to Taiwan from southern China in the late Ming Dynasty by the Han people approximately 400 years ago (DAFTPG 1989). Two Wu-K’o (Chiayi Wu-K’o, Taipei Woo-Co in IRRI) accessions are included in the 3K dataset: a Wu-Chien (Taichung Woo Gen 2) accession and a Wu-Li (Taitung Woo-Li in IRRI) accession. However, different institutes use different spellings for these accessions, such as Wu-Chien in TARI versus Woo Gen in IRRI, and in some cases one institute even uses different spellings for the same accession, such as both Wu-K’o and Woo-Co in IRRI. In summary, the Chinese word for Woo or Wu is exactly the same, but it is spelled differently by various institutes. Likewise, the terms Gen and Chien rendered in the Roman alphabet for use in English refer to the same Chinese word. Table S16 lists the Chinese and English names of these lines, including their accession numbers in different seed stock centers. This is important information for rice researchers because it may help eliminate the misuse of genetic resources.

### Many modern *indica* varieties have functional nucleotide polymorphisms in *SD1*

Most *japonica* rice cultivars, including traditional and modern accessions, are shorter than *indica* landraces due to the *GR*-to-*EQ* allele variation. Such effects are reflected in modern breeding programs. Notably, few modern *japonica* varieties have LOF mutation of *SD1* alleles. Approximately 60 *japonica* accessions in the 3K panel contain *sd1* types 3–6, with these 4 alleles originating in *japonica* rice. In Taiwan, both *japonica* and *indica* rice varieties are produced and consumed, and *japonica* ⋅ *indica* crosses have often been used in breeding programs. However, no modern *japonica* varieties harbor the *DGWG* (type 7) mutation, despite the *indica* parental lines used in breeding carrying this allele. Thus, plant height was not a major target during the breeding of most *japonica* varieties. By contrast, all modern *indica* rice varieties contain a type 7–10 *sd1* allele, and more than 400 (35%) modern varieties in the 3K dataset carry the 383-bp deletion. Thus, the *sd1* type 7 allele has been used intensively in breeding programs worldwide, where the semi-dwarf trait was predominantly derived from DGWG or its progeny (Khush et al. 2001; Prasad et al. 2001). Approximately 40 varieties contain *sd1* types 8 or 9, mostly modern varieties in China. Type 10, the allele from Indonesia, was used in ~ 50 accessions in Southeast Asia. Notably, *indica* rice production comprises ~ 80% of worldwide rice production. The mutated *SD1* gene is one of the primary reasons for the increase in rice production since the mid-1960s because it leads to semi-dwarf growth habit resulting in lodging resistance and the ability to withstand heavy fertilizer applications.

### Functional implications of *SD1* haplotypes and applications in genome editing

We observed that plant height varied among *SD1* haplotypes (Fig. 2), consistent with the findings of Ogi et al. (1993). Using isogenic lines of the *japonica* variety ‘Norin 29’, they demonstrated the functional effects of four *sd1* alleles on culm length, internode length, and yield-related traits. When compared with Norin 29 (85.1 cm), SC-TN1 (*DGWG* type) showed the greatest reduction in culm length (57.5 cm), whereas SC-AJNT (*Aijiao-Nante* type) and SC-SRN (*Jikkoku* type) exhibited a less severe reduction (60.4 cm and 59.4 cm, respectively). (Ogi et al. 1993). Furthermore, Nakamura et al. (1991) analyzed protein profiles in germinating seeds of these lines and identified differential protein spots: SRP-1 in Norin 29 and SRP-2 in SC-TN1. This suggests that the *sd1* mutation alters downstream protein expression (Nakamura et al. 1991). In this study, we classified the 12 *SD1* haplotypes based on their mutation sites (Table 1), and we observed distinct differences in plant height among the haplotype groups (Fig. 2). The protein modeling results (Fig. 5) further suggested that the positions of these mutations have distinct impacts on *GA*_20_*ox*-2 protein structure and function.

While natural *sd1* alleles have been extensively characterized, recent advancements have enabled the artificial generation of these traits. Two recent studies used CRISPR-Cas9 to generate semi-dwarf rice lines and analyzed the agronomic traits of the edited plants (Hu et al. 2019; Biswas et al. 2020). Hu et al. (2019) targeted the *SD1* gene in two elite landraces, Kasalath (*aus*) and TeTePu. Using 10 or 30 randomly chosen T_2_ plants grown in paddy fields under natural conditions, the authors demonstrated that gene editing could rapidly create *sd1* mutants. The seed production of the edited plants was better than that of the wild type under modern cultivation, while the desirable agronomic traits of these varieties (i.e., tolerance for low phosphorus in Kasalath and broad-spectrum resistance to diseases and insects in TeTePu) were maintained (Hu et al. 2019).

In contrast, Biswas et al. (2020) edited *SD1* in three elite cultivars: 9815B, JIAODA138, and HUAIDAO1055. The authors investigated plant height and yield across the T_3_ and T_4_ generations using 10 independent lines (10 plants each) grown in the paddy field under natural conditions. The mutated plants showed significantly reduced seed production even though they had reduced plant height. Therefore, the authors suggested that the use of the CRISPR-Cas9 system to edit *SD1* can have varying results and still requires more effort and time (Biswas et al. 2020). Considering that Biswas et al. (2020) utilized advanced generations (T_3_/T_4_), a larger number of independent lines, and more comprehensive statistical analysis, their results may provide a more robust assessment than the earlier findings of Hu et al. (2019).

Given the variable outcomes observed in previous studies, selecting the optimal target site is crucial for successful genome editing. Moreover, different *sd1* alleles result in varying phenotypic effects. The results of our SD1 protein modeling should therefore be valuable in providing insights for rational design, shedding light on how to precisely manipulate plant height through *SD1* modification.

### The impact of DGWG on the Green Revolution

Prior to the release of IR8, the earliest semi-dwarf modern rice variety was TN1. Derived from a cross between DGWG and Tsai-Yuan-Chung performed during the second cropping season of 1949, the line designated as “Taichung Native Bred 1” was selected during the second season in 1952. It was officially released as TN1 in early 1957 and subsequently cultivated widely in Taiwan. Its full genome is available (Panibe et al. 2021). In 1966, 50 tons of TN1 seeds were shipped from Taiwan to India due to a local famine. Notably, TN1 covered a production area of 800,000 ha in India during 1968–1969. Taiwanese semi-dwarf *indica* germplasm served as the foundation for new varieties subsequently developed at the IRRI and elsewhere in Asia (Barker et al. 1985; Hargrove et al. 1988).

Following the era of TN1, IR8 was developed from a cross between Peta and DGWG in 1962. A single plant (the 3rd plant of line 288) in the F_5_ generation was selected to produce F_6_ (IR8-288-3) seed and was sent for testing without further selection at the time. In 1966, IR8-288-3 showed dramatic yield increases in the trials and was later named IR8 in the same year (Khush et al. 2001). Khush et al. (2001) reported that IR8 was photoperiod insensitive and matured in 130–135 days; thereafter, the IRRI devoted efforts to producing a uniform seed source of IR8.

However, this duration was not short enough to allow an additional crop season within a year. Therefore, the IRRI continued to improve the rice lines, aiming for a shorter growth duration. Subsequently, IR28 and IR36 matured in 110 days, and the growth duration for IR50 and IR58 was further reduced to 100–105 days (Khush 1987; Khush et al. 2001). Our results demonstrate that the majority of modern varieties possessing the LOF *sd1* allele also carry the LOF *hd1* allele, particularly in the three major indica-producing countries: India, Indonesia, and the Philippines (Table S14). This confirms the continuous selection for early maturity in rice breeding.

Most Green Revolution rice varieties are of the *indica* type and are grown in subtropical and tropical regions, where temperatures are suitable for plant growth year-round. Thus, changes in sensitivity to day length play an important role in their cultivation because this can increase the number of cropping seasons per year and adjust the growing season to escape from or avoid stress caused by seasonal typhoons, monsoons, or drought. As a result of combining the DGWG-derived semi-dwarf trait with photoperiod-insensitivity traits, rice production has increased tremendously since the mid-1960s.

## Conclusions

This study provides a comprehensive overview of natural variation in *SD1*, revealing 12 haplotypes with distinct evolutionary histories and functional impacts. The GR-to-EQ amino acid changes were confirmed as ancient domestication signatures that became fixed in most traditional *japonica* landraces, whereas other *sd1* mutations arose more recently. In particular, the 383-bp DGWG deletion was preferentially incorporated into modern *indica* cultivars during the Green Revolution. We also show that the loss-of-function *hd1* allele from DGWG was introgressed into IR8 and many subsequent *indica* varieties, indicating that photoperiod insensitivity was selected alongside reduced plant height to enable early maturity and multiple cropping. Together, our findings clarify the dual genetic foundations of the Green Revolution and provide an evolutionary and functional framework that can guide future rice breeding and genome-editing strategies.

## Materials and methods

### Classification of *SD1* haplotypes and DNA sequence analysis

To identify additional *SD1* haplotypes from the public database, two whole-genome sequencing datasets were used: the Rice 3K project (3K-RGP 2014) and an Asian wild rice dataset (Zhao et al. 2018). Some Taiwanese landraces were also sequenced for comparison. Information including the accession names, seed code numbers, *sd1* types, and DNA accession numbers is provided in Table S8. The paired reads were mapped against the Nipponbare IRGSP-1.0 RefSeq database (Kawahara et al. 2013; IRGSP 2005). Each VCF file of the 3K sequences was first filtered by read depth > 5 and SNP frequency > 0.8. To examine the functional impact of nucleotide variants, rice genome sequencing data were analyzed using SnpEff (Cingolani et al. 2012). Based on genome annotation, these sequence variants were classified according to their location (ORF, intron, and splice sites) and predicted functional impact (missense mutation, frame shift, and early translational termination).

Information in the SAM format was used to determine whether the paired reads were properly aligned based on insert size and orientation. The larger insert size of properly oriented paired reads indicates a possible deletion between the paired reads. To illustrate that the long deletion occurred at exons or the promoter region, snapshot images were produced using Integrative Genomics Viewer (Robinson et al. 2011; Thorvaldsdottir et al. 2013; Robinson et al. 2017) for the variant event in the next-generation sequencing mapped images of the genome for the indicated lines. Accessions carrying exclusively type 1 or type 2 mutations were first identified. Subsequently, types 3 through 12 were classified, as they may share amino acid substitutions at positions 100 or 340 with types 1 and 2.

### Estimating the diversity of different *SD1* haplotypes

DNA sequences were aligned using MUSCLE (Edgar 2004a, b). The − 10- to + 10-kb region of the *SD1* gene, corresponding to Nipponbare genome chromosome 1 positions 38,372,466 to 38,395,208, was used for analysis. Statistical analysis was performed using DnaSP.v6 (Rozas et al. 2017). Items analyzed included the number of polymorphic (segregating) sites (*S*); total number of mutations (eta, *η*); average number of nucleotide differences (*k*); nucleotide diversity (*π*; Tajima 1983); theta (*θ*; per sequence) from eta (*η*); and Watterson’s estimator of *θ* (per site) from *η* (*θw*; Watterson 1975). The Tajima’s *D* neutrality test (Tajima 1989) was used to test the neutral mutation hypothesis. The *D* value is based on the discrepancy between *π* and *θw*. Thus, negative values indicate excess low-frequency polymorphism. These values were calculated after removing missing data and alignment gaps.

### Phylogenetic analysis of *SD1*

Phylogenetic relationships were inferred from nucleotide sequences derived from the selected VCF regions. The VCF files were converted into PHYLIP format using vcf2phylip v2.6 (Ortiz 2019). The evolutionary history was reconstructed using the Neighbor-Joining (NJ) method (Saitou and Nei 1987). Evolutionary distances were calculated via the Maximum Composite Likelihood method (Tamura et al. 2004), expressed as the number of base substitutions per site. To ensure data robustness, the pairwise deletion option was applied to all ambiguous positions for each sequence pair in the final analysis. Statistical support for internal nodes was assessed through a bootstrap test with 1,000 replicates (Felsenstein 1985). In the final phylogenetic trees, bootstrap values lower than 60% were collapsed to prioritize well-supported clades. All computational procedures were performed in MEGA12 (Stecher et al. 2020, 2026) using multi-threaded parallel processing to enhance computational efficiency.

### Protein modeling of GA_20_ox-2

A crystal structure of the gene product of SD1 (i.e., GA20ox-2) is not available. Therefore, a homology model was generated using the MODELLER module (Webb and Sali 2021) implemented in the Chimera software package (Pettersen et al. 2004). The crystal structure of GA_2_ox3 (PDB ID: 6KU3; Takehara et al. 2020) was used as the template because this enzyme utilizes a substrate (GA_4_) that is structurally similar to GA_20_. The quality of the resulting GA_20_ox-2 model was assessed using the GA341 score. According to Chimera, the GA341 score reflects model reliability; a score > 0.7 indicates a probability of correct folding greater than 95% (Melo et al. 2002). The GA_20_ox-2 model yielded a GA341 score of 1.0, suggesting high reliability. For docking, the modeled GA_20_ox-2 structure was prepared using the Protein Preparation Wizard module of Maestro (Schrödinger), which added hydrogen atoms and assigned atomic charges. The compounds GA_20_ and succinate were prepared using the LigPrep Wizard module of Maestro (Schrödinger). Succinate was first docked into the binding site using Glide XP (Friesner et al. 2006). The docked position of succinate was considered part of the active site, after which GA_20_ was docked into the same binding site.

## Electronic Supplementary Material

Below is the link to the electronic supplementary material.

Supplementary Material 1: Table S1. Accession numbers, accession names, collection regions, subspecies, and types of SD1 alleles in the 3K dataset

Supplementary Material 2: Table S2. ANOVA for plant height of the 11 SD1 haplotypes. Table S3. Statistical analysis of plant height for the SD1 haplotypes. Table S4. Distribution of GR, EQ, and ER at aa positions 100 and 340 in the 3K panel. Table S5. Accessions used for phylogenetic analysis of type 7 sd1 alleles. Table S6. Accessions used for phylogenetic analysis of type 6 sd1 alleles. Table S7. Accessions used for phylogenetic analysis of types 6 and 10 sd1 alleles. Table S8. Taiwanese rice landraces used in this study. Table S9. Accessions used for phylogenetic analysis of type 7 SD1 genes. Table S10. Accessions used for phylogenetic analysis of 67 accessions containing type 7 sd1 alleles. Table S11. Accessions used for phylogenetic analysis of 10 Taiwanese and wild rice accessions harboring type 7 sd1 alleles. Table S12. Accessions used to calculate the selection parameters for type 2 SD1 genes. Table S13. Selection parameters for 4 SD1 haplotypes. Table S14. Distribution of hd1 type 7 and type 13 alleles among 3K accessions with loss-of-function sd1 mutations in Asian rice-growing countries. Table S15. Gene IDs, Plant Ontology (PO) and Trait Ontology (TO) annotations of GA20ox homologs in rice. Table S16. Dee-Geo-Woo-Gen (DGWG)-related accessions and their spellings in seed stock centers

Supplementary Material 3: Fig. S1. Types 6 and 7 sd1 alleles shown in Integrative Genomics Viewer images. Fig. S2. Geographic distribution of accessions harboring type 6 sd1 alleles. Fig. S3. Phylogenetic analysis of types 6 and 7 sd1 alleles. Fig. S4. Phylogenetic analysis of accessions harboring type 6 sd1 alleles. Fig. S5. Phylogenetic analysis of accessions harboring types 6 and 10 sd1 alleles. Fig. S6. Phylogenetic analysis of 67 accessions harboring type 7 sd1 alleles. Fig. S7. Phylogenetic analysis of 10 accessions harboring type 7 sd1 alleles. Fig. S8. Venn diagram of single-nucleotide polymorphisms (SNPs) shared among five early landraces with type 7 sd1 mutations. Fig. S9. Alignment of GA20 oxidase homologs in the rice genome

## Data Availability

All data generated or analyzed during this study are included in this article and the supplementary information files. All seeds mentioned are available in the TARI or IRRI seed stock centers.

## Abbreviations

DGWG: Dee-Geo-Woo-Gen
Indel: Insertion and deletion
IRRI: International Rice Research Institute
LOF: Loss of function
NPGRC: National Plant Genetics Resource Center
NGS: Next-generation sequencing
ORF: Open reading frame
QTL: Quantitative trait loci
TARI: Taiwan Agricultural Research Institute
TN1: Taichung Native 1
TR: Tandem repeat
