# Supplementary material for: Both semi-dwarf and photoperiod-insensitive traits in rice were important for the Green Revolution
Source: Bot Stud. 2026 Mar 26;67:6. doi: 10.1186/s40529-026-00493-3 (PMC13022152; doi:10.1186/s40529-026-00493-3)
Supplement: Supplementary file 3 — Supplementary Material 3: Fig. S1. Types 6 and 7 sd1 alleles shown in Integrative Genomics Viewer images. Fig. S2. Geographic distribution of accessions harboring type 6 sd1 alleles. Fig. S3. Phylogenetic analysis of types 6 and 7 sd1 alleles. Fig. S4. Phylogenetic analysis of accessions harboring type 6 sd1 alleles. Fig. S5. Phylogenetic analysis of accessions harboring types 6 and 10 sd1 alleles. Fig. S6. Phylogenetic analysis of 67 accessions harboring type 7 sd1 alleles. Fig. S7. Phylogenetic analysis of 10 accessions harboring type 7 sd1 alleles. Fig. S8. Venn diagram of single-nucleotide polymorphisms (SNPs) shared among five early landraces with type 7 sd1 mutations. Fig. S9. Alignment of GA20 oxidase homologs in the rice genome [file 40529_2026_493_MOESM3_ESM.docx]

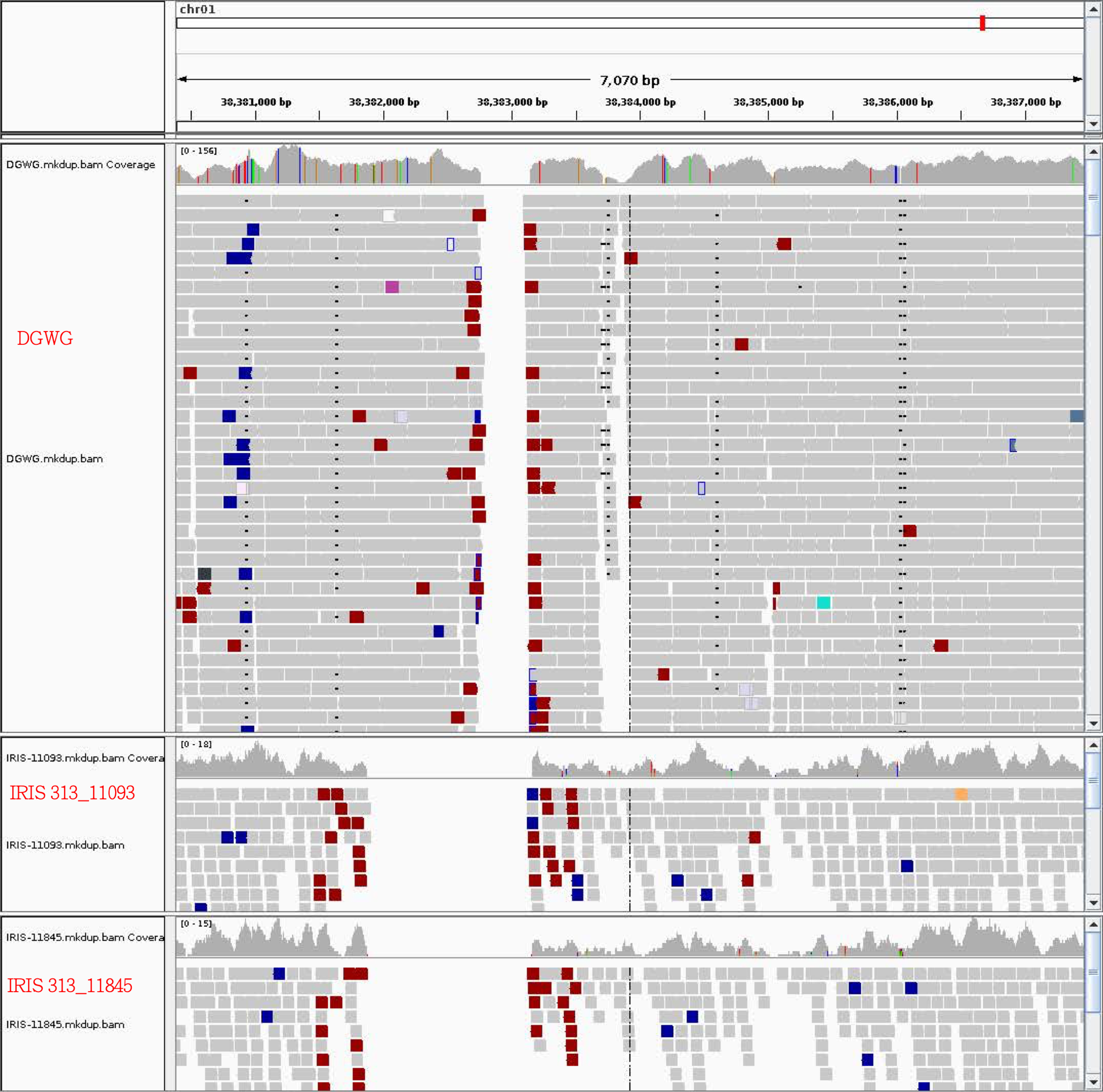


Fig. S1. Types 6 and 7 *sd1* alleles shown in Integrative Genomics Viewer images.

The long type 7 (DGWG) and type 6 (IRIS 313-11093 and IRIS 313_11834) deletions are shown. Alignments to Nipponbare IRGSP 1.0 are represented by gray rectangles and mismatched reads by red or blue rectangles.


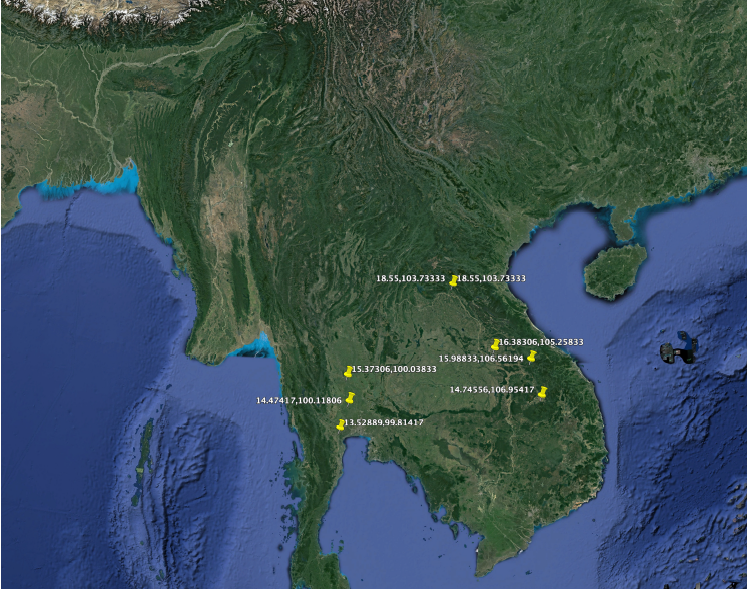


Fig. S2. Geographic distribution of accessions harboring type 6 *sd1* alleles.

Ten traditional accessions are from Laos and Thailand. In some cases, more than one accession was collected from the same GPS location.


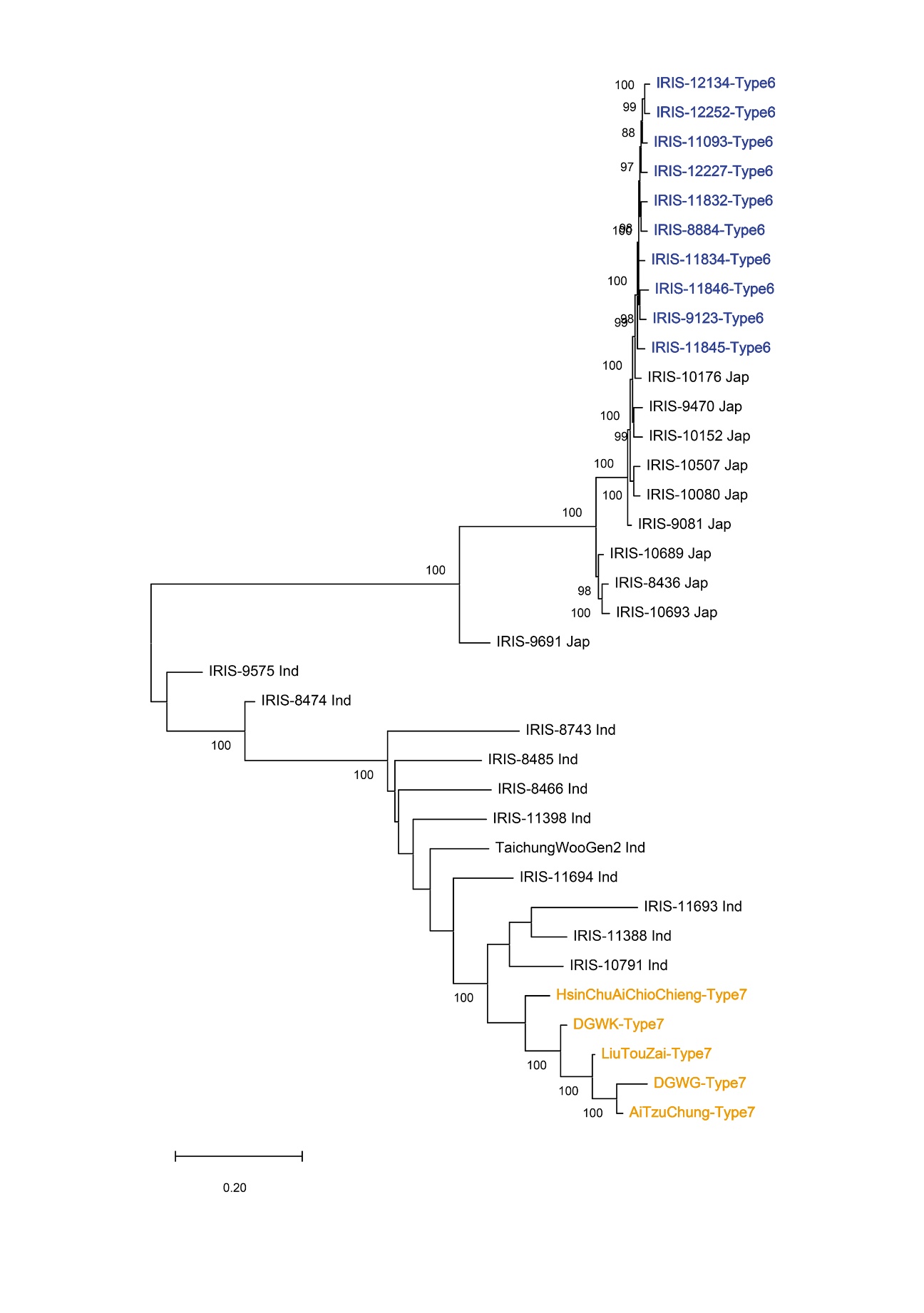


Fig. S3. Phylogenetic analysis of types 6 and 7 *sd1* alleles.

Nucleotide changes in landraces with type 6 (highlighted in blue) and 7 (in orange) nucleotide changes in the region from –1 to + 1 Mb in *SD1*. Some *indica* and *japonica* accessions (in black) were also analyzed for comparison. Numbers at nodes indicate bootstrap support values (1,000 replicates). Table S5 lists the accessions used.


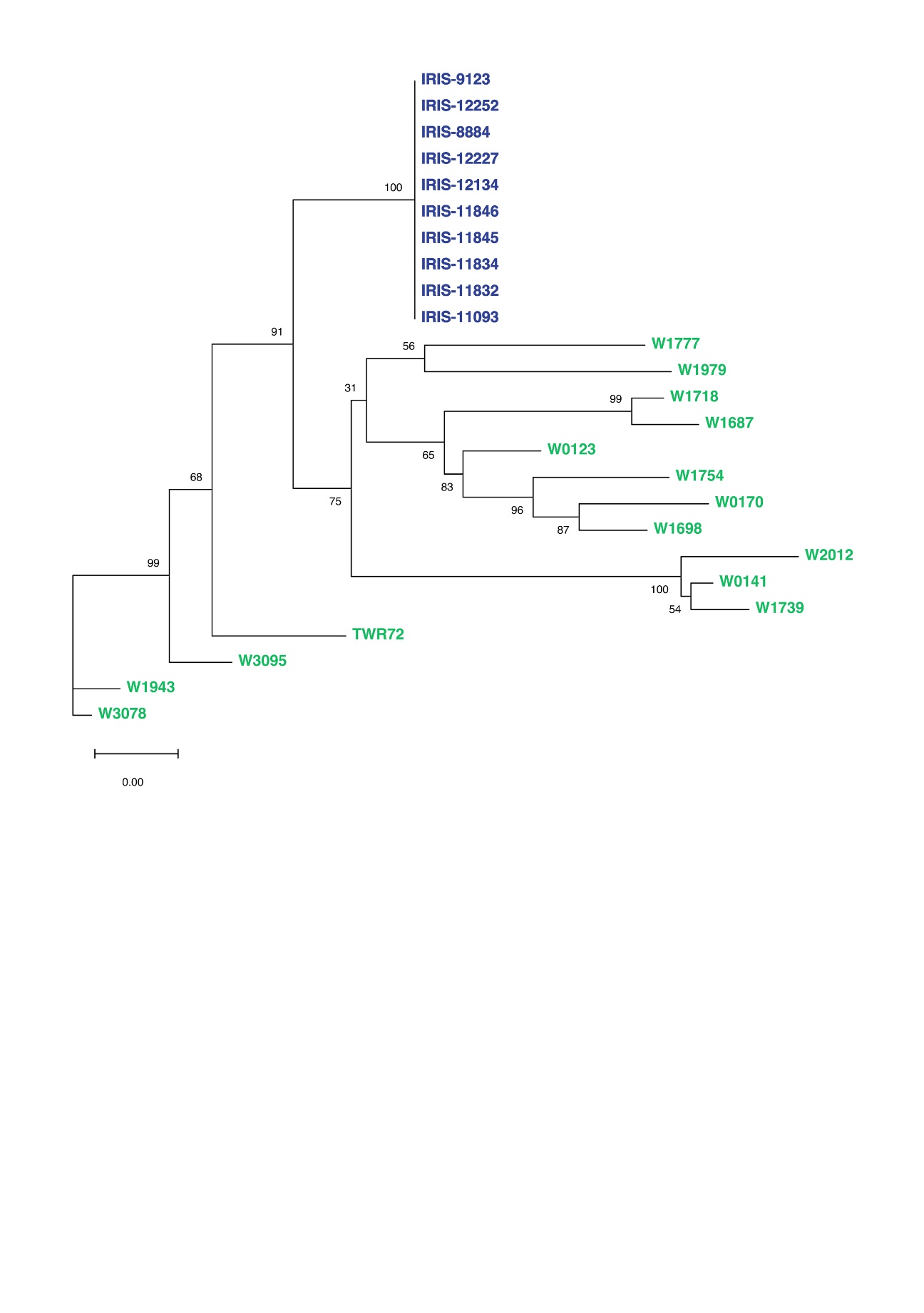


Fig. S4. Phylogenetic analysis of accessions harboring type 6 *sd1* alleles.

Accessions with type 6 nucleotide changes in the region from –1 to +1 kb in *SD1* (highlighted in blue) and wild rice (in green) were used. Numbers at nodes indicate bootstrap support values (1,000 replicates). Table S6 lists the accessions used.


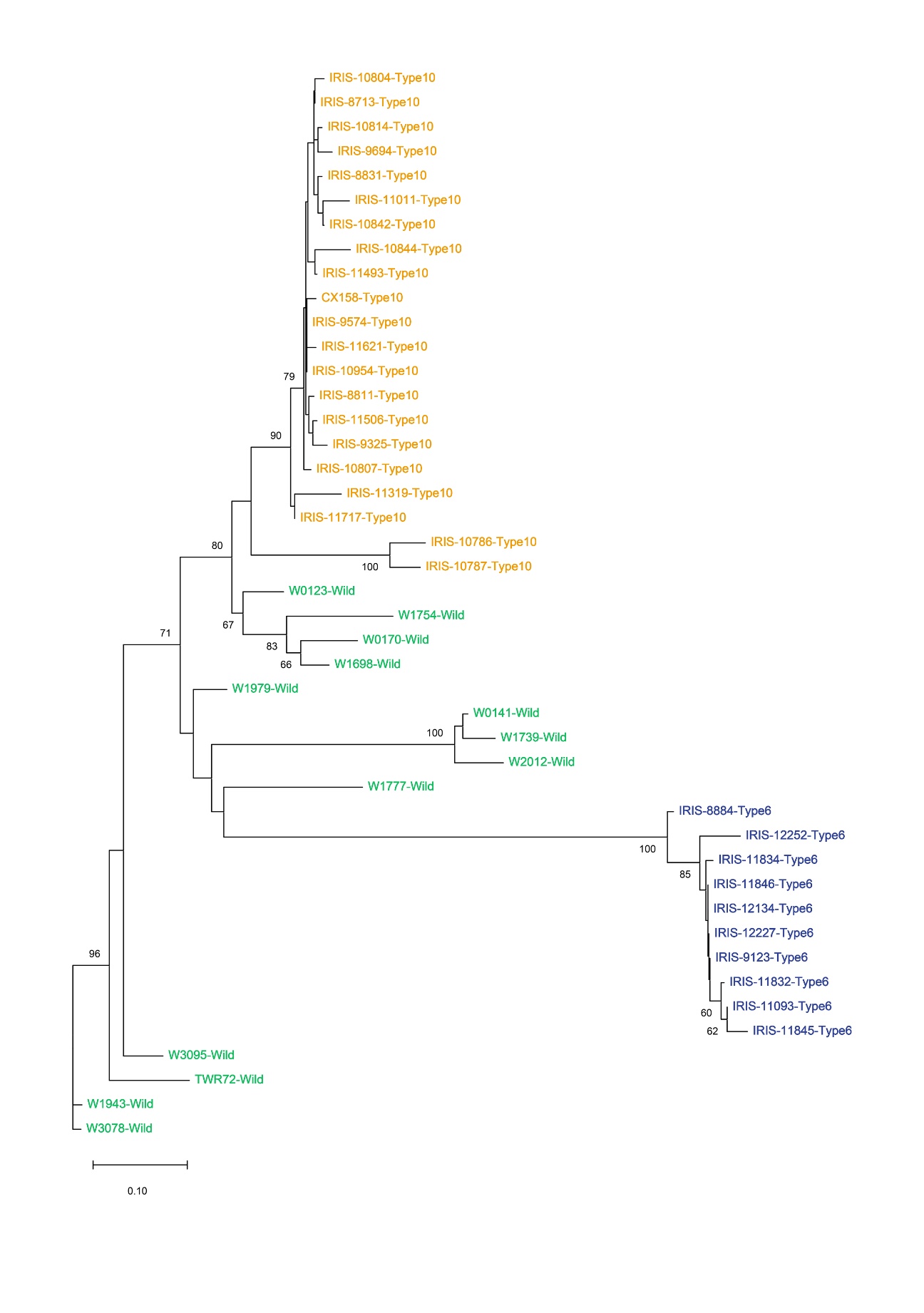


Fig. S5. Phylogenetic analysis of accessions harboring type 6 and 10 *sd1* alleles.

Landraces with type 6 (highlighted in blue) and 10 (in orange) nucleotide changes in the region from –1 to + 1 kb in *SD1* were used. Some Asian wild rice accessions are highlighted in green. Numbers at nodes indicate bootstrap support values (1,000 replicates). Table S7 lists the accessions used.

| **A**  **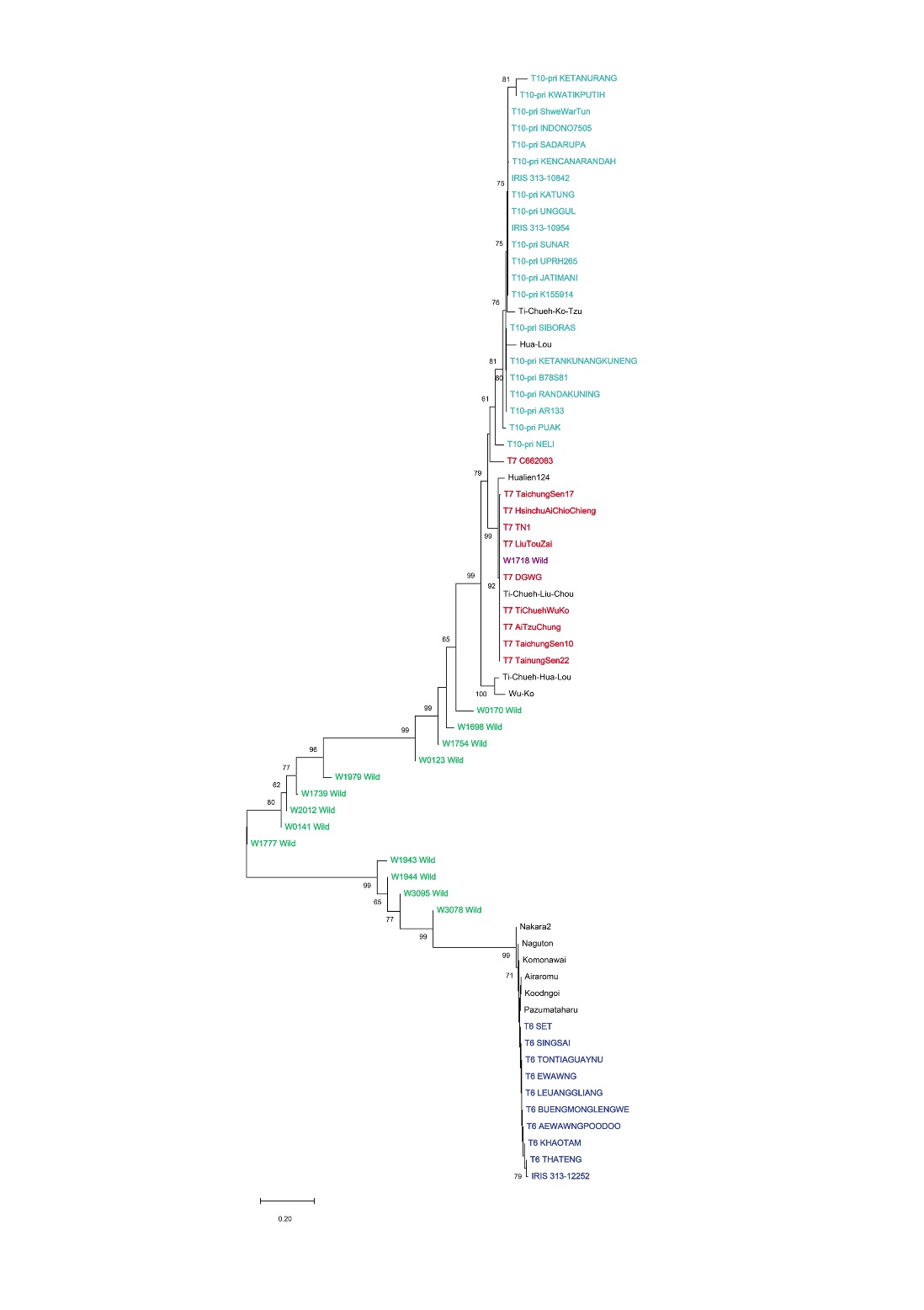** | **B**  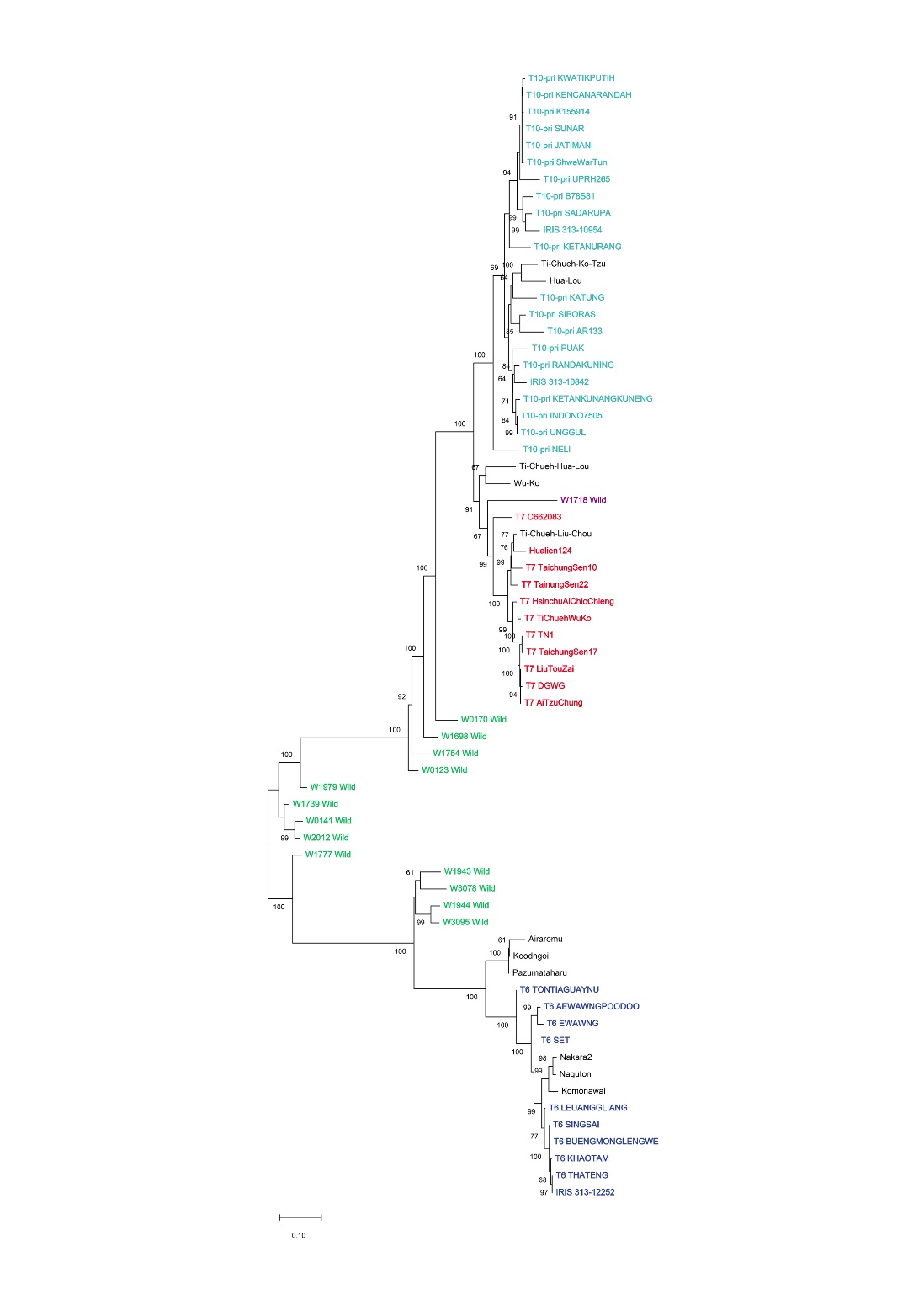 |
| --- | --- |
| **C**  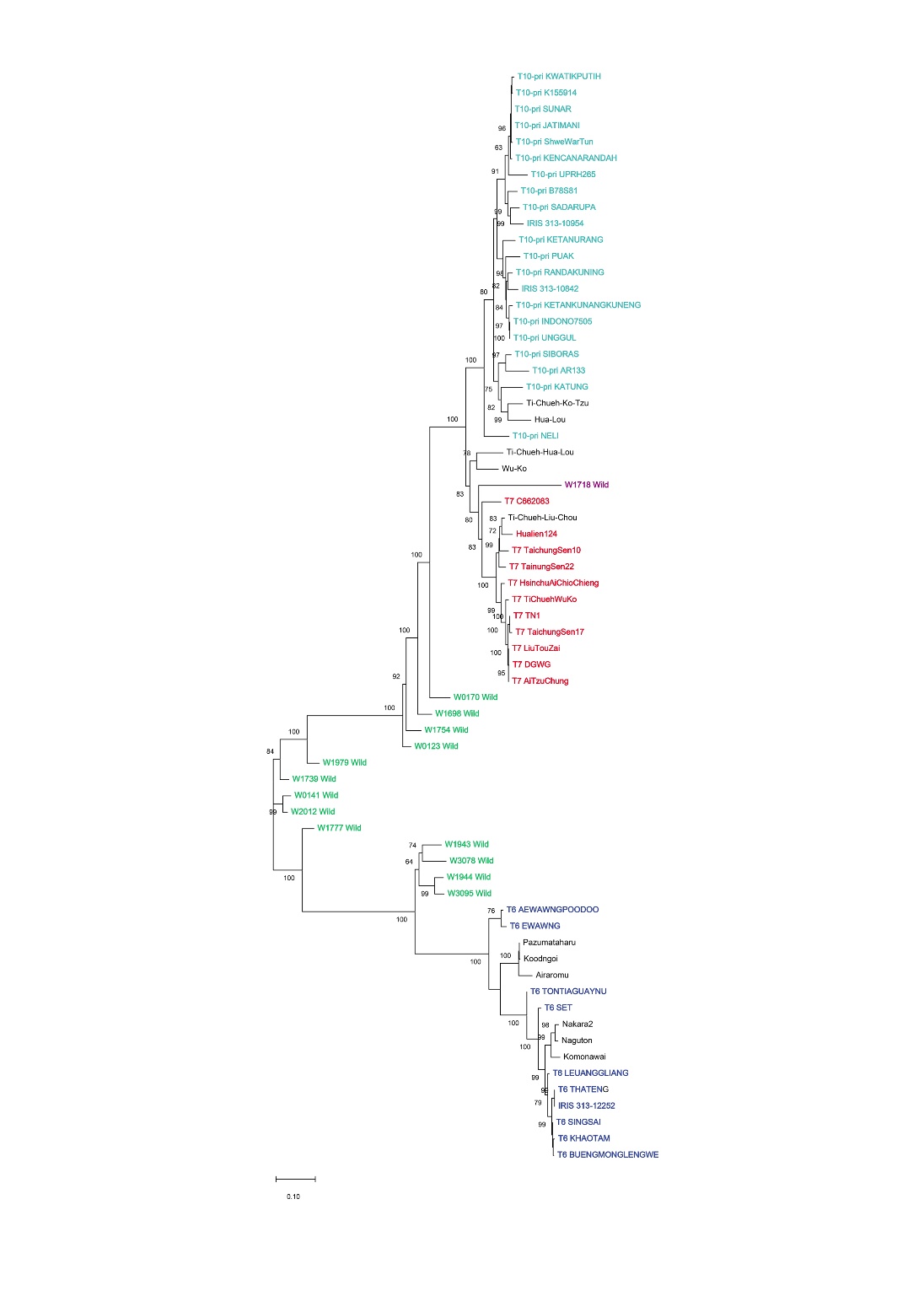 | **D**  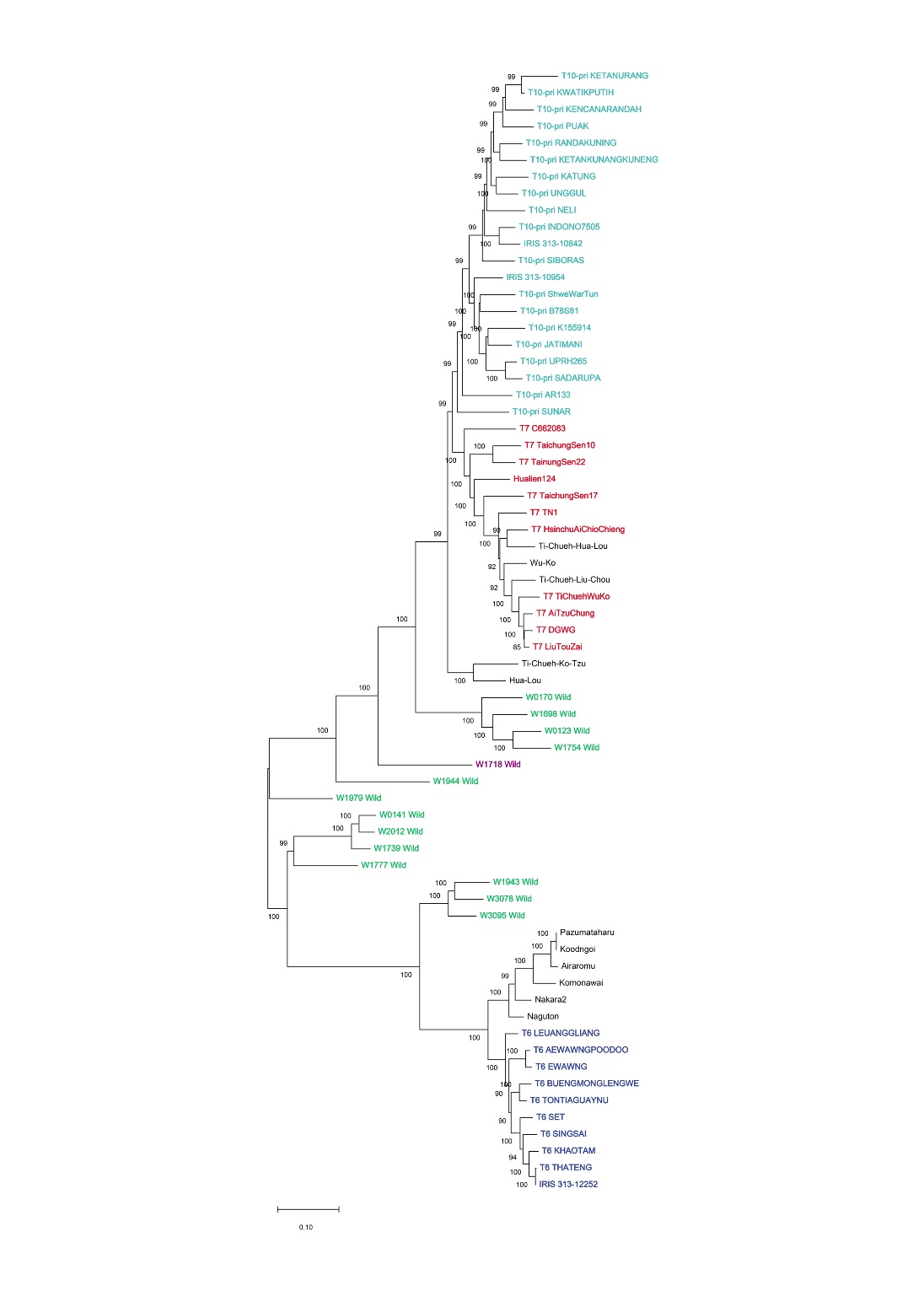 |

Fig. S6. Phylogenetic analysis of 67 accessions harboring type 7 *sd1* alleles.

Nucleotide changes in *SD1* regions –1 to +1 Mb (panel A), –9 to + 9 Mb (panel B), and –10 to +10 Mb (panel C) and whole-genome sequencing information (panel D). Blue indicates landraces with the type 6 *SD1* mutation, red indicates landraces and some modern varieties with the type 7 mutation, cyan indicates landraces with the type 10 mutation, green indicates wild rice accessions, and purple indicates the wild rice accession W1718. Black indicates other Taiwanese landraces without the type 7 mutation. Numbers at nodes indicate bootstrap support values (1,000 replicates). Accession information is provided in Table S10.


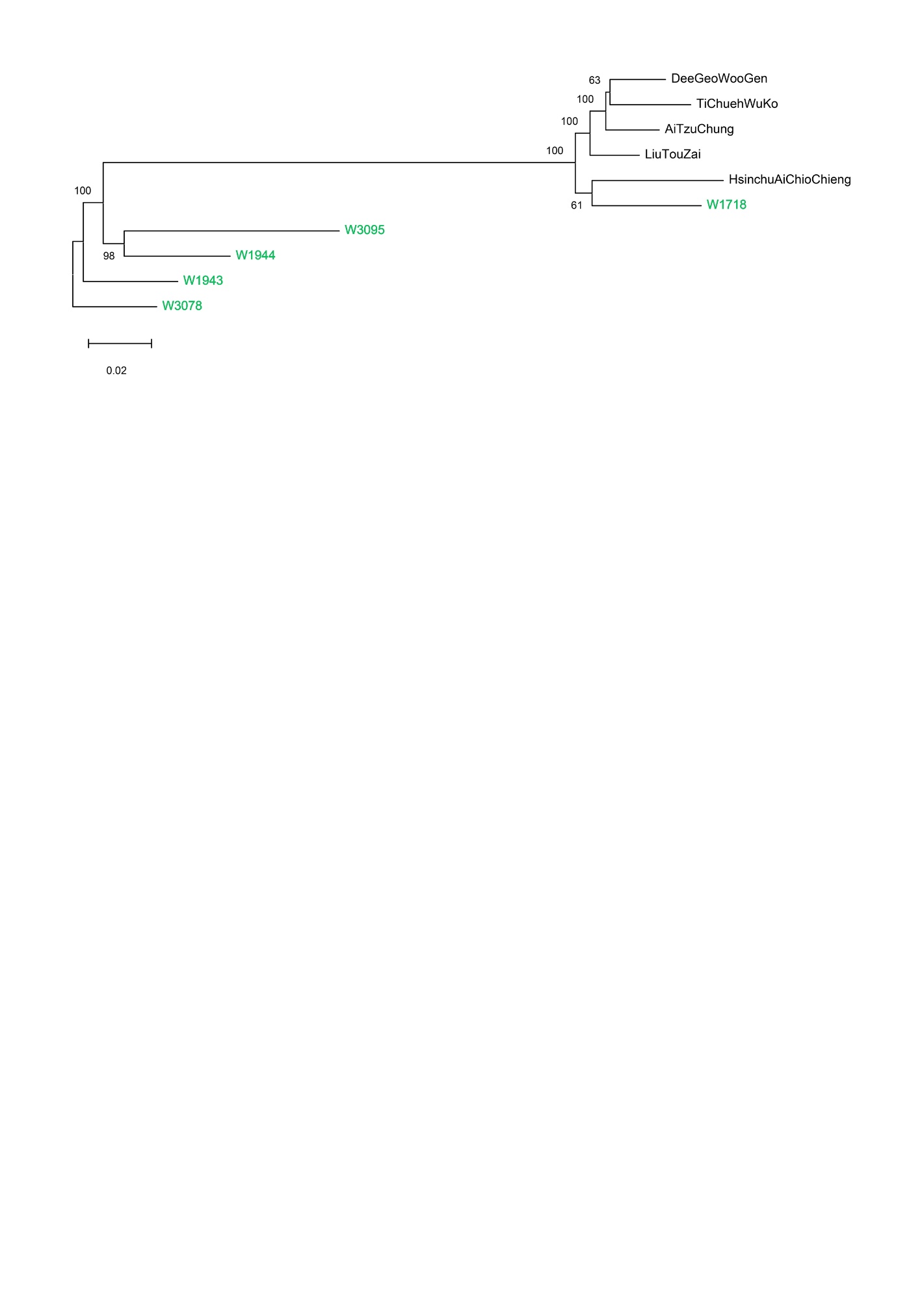


Fig. S7. Phylogenetic analysis of 10 accessions harboring type 7 *sd1* alleles.

Nucleotide changes in the –200 to +200-kb region of *SD1* in the five Taiwanese landraces carrying type 7 mutations and five wild rice accessions collected in southern China were used for comparison. Numbers at nodes indicate bootstrap support values (1,000 replicates). Accession information is provided in Table S11.


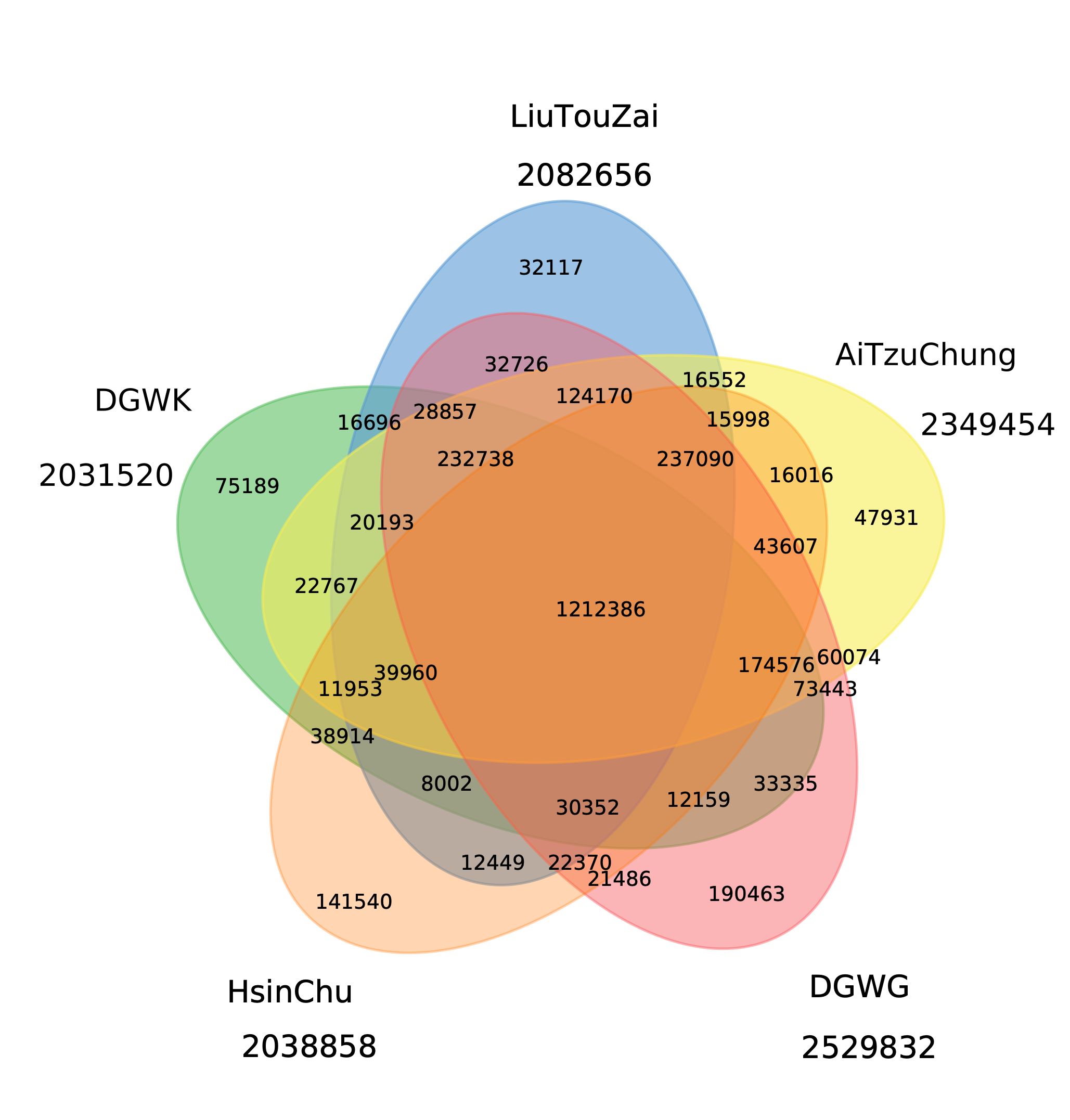


Fig. S8. Venn diagram of single-nucleotide polymorphisms (SNPs) shared among five early landraces with type 7 *sd1* mutations. The lines were Dee-Geo-Woo-Gen (DGWG), Ti-Chueh-Wo-Ko (DGWK), Ai-Tzu-Chung, Hsinchu-Ai-Chueh-Chien (HsinChu), and Liu-Tou-Tzu (LiuTouZai).





Fig. S9. Alignment of GA_20_ oxidase homologs in the rice genome.

Os01t0883800 is SD1 protein, with Kasalath aa residues instead of Nipponbare because *indica* rice is the wild type. 1 indicates Gly94 mutated to Val in type 3; 2, Pro240 to Leu in type 10; 3, Leu266 to Phe in type 4; and 4, Asp349 to His in type 5. a indicates Gly100/Arg340 mutated to Glu100/Gln340 in type 2.
